# Proteome Differences in Smooth Muscle Cells from Diabetic and Non-Diabetic Abdominal Aortic Aneurysm Patients Reveal Metformin-Induced Mechanisms

**DOI:** 10.3390/medsci13030184

**Published:** 2025-09-10

**Authors:** Tara A. R. van Merrienboer, Karlijn B. Rombouts, Albert C. W. A. van Wijk, Jaco C. Knol, Thang V. Pham, Sander R. Piersma, Connie R. Jimenez, Ron Balm, Kak K. Yeung, Vivian de Waard

**Affiliations:** 1 Department of Surgery, Amsterdam UMC location University of Amsterdam, Meibergdreef 9, 1105 AZ Amsterdam, The Netherlands; a.c.wijk@amsterdamumc.nl (A.C.W.A.v.W.); r.balm@amsterdamumc.nl (R.B.); k.yeung@amsterdamumc.nl (K.K.Y.); 2 Department of Medical Biochemistry, Amsterdam UMC location University of Amsterdam, Meibergdreef 9, 1105 AZ Amsterdam, The Netherlands; v.dewaard@amsterdamumc.nl; 3 Amsterdam Cardiovascular Sciences, Atherosclerosis and Aortic Diseases, Amsterdam UMC location University of Amsterdam, Meibergdreef 9, 1105 AZ Amsterdam, The Netherlands; k.b.rombouts@amsterdamumc.nl; 4 Department of Physiology, Amsterdam UMC location Vrije Universiteit Amsterdam, De Boelelaan 1117, 1081 HV Amsterdam, The Netherlands; 5 OncoProteomics Laboratory, Medical Oncology, Amsterdam UMC location Vrije Universiteit Amsterdam, Van der Boechorststraat 6, 1081 BT Amsterdam, The Netherlands; j.knol@amsterdamumc.nl (J.C.K.); t.pham@amsterdamumc.nl (T.V.P.); s.piersma@amsterdamumc.nl (S.R.P.); c.jimenez@amsterdamumc.nl (C.R.J.); 6 Proteomics Core Resource, Amsterdam UMC location Vrije Universiteit Amsterdam, Van der Boechorststraat 6, 1081 BT Amsterdam, The Netherlands

**Keywords:** abdominal, aortic aneurysm, proteomics, redox, antioxidants, smooth muscle cell, metformin

## Abstract

**Aims**: Surgery remains the only definitive treatment option for abdominal aortic aneurysms (AAA), as no conclusive evidence supports drug effectiveness in preventing AAA growth. Although type 2 diabetes (T2D) is an important cardiovascular risk factor, patients with T2D show reduced AAA presence and growth, associated with metformin use. We aimed to investigate the potential benefits of metformin on AAA using proteomics and in vitro experiments. **Methods**: Proteomics analysis using tandem mass spectrometry was performed on aortic smooth muscle cells (SMCs) from non-pathological controls (C-SMC, *n* = 8), non-diabetic (ND, *n* = 19) and diabetic (D, *n* = 5) AAA patients. Key findings were subsequently validated in aortic tissue using mass spectrometry-based proteomics. SMCs were cultured with/without metformin and analyzed. **Results**: Comparison of the proteome of SMCs from ND-AAA patients with controls revealed a reduction in proteins associated with metabolic processes and mitochondrial function. Cytoskeletal and extracellular matrix (ECM) proteins were elevated in ND-AAA-SMCs versus C-SMCs, with a similar cluster of mechanosensitive proteins being increased in ND-AAA-SMCs versus D-AAA-SMCs. D-AAA-SMCs showed an improved metabolic and antioxidant profile, enriched in pentose phosphate pathway proteins responsible for NAD(P)H generation (G6PD, PGD) and NAD(P)H-dependent antioxidants (NQO1, CBR1, AKR1C1, AKR1B1, GSTM1), all regulated by NRF2, an antioxidant transcription factor. Over half of the proteins identified in the protein–protein interaction network, constructed from proteins with higher expression in D-AAA SMCs versus ND-AAA SMCs, were verified in D-AAA aortic tissue. In vitro, metformin causes a shift from aerobic to anaerobic metabolism, increased AMPK activation and elevated mitochondrial biogenesis, indicated by increased *PGC-1α* expression. Metformin increased the gene expression of *PGD*, *CBR1* and the protein expression of NQO1, with enhanced translocation of pNRF2 to the nucleus, due to reduced KEAP1 as negative regulator of NRF2. Consequently, metformin enhanced the gene expression of well-known antioxidant regulators *SOD2* and *CAT*. **Conclusions**: This study identified significant differences in the proteome of SMCs derived from controls, ND-AAA and D-AAA patients. It highlights distinct pathways in relation to mechanosensing, metabolism and redox balance as therapeutic targets of metformin that may underlie its inhibition of AAA progression.

## 1. Introduction

An abdominal aortic aneurysm (AAA) is a pathological dilatation of the aortic wall, defined as an enlargement of at least 50% beyond the normal aortic diameter [1]. It is a life-threatening condition due to the risk of rupture. If an AAA ruptures, only one-third of the patients reach the hospital on time for surgical treatment, and the mortality rate is up to 80% [2]. AAA is usually asymptomatic and often remains undetected until it is incidentally discovered during imaging for other indications. The only definitive treatment options are open repair or endovascular aneurysm repair (EVAR) [3]. Surgical treatment is recommended for an AAA that becomes symptomatic or surpasses 5.5 cm in males or 5.0 cm in females [4]. A conservative follow-up approach is recommended if the aneurysm remains below these thresholds. Currently, there is no pharmacological therapy since there is still no conclusive evidence supporting drug effectiveness in preventing AAA growth [5].

Although type 2 diabetes (T2D) is a well-known risk factor for cardiovascular disease, many studies have shown consistently that patients with T2D are less likely to develop AAA and experience AAA growth and rupture [3,6,7]. One proposed explanation is the use of metformin, the first-line treatment for T2D. According to a recent systematic review and meta-analysis, metformin use was associated with a 0.73 mm/year slower growth rate of AAA compared to non-users [5].

Smooth muscle cells (SMC) are the most abundant cell type in the aortic wall and SMC dysfunction plays a crucial role in the pathophysiology of AAA. In our previous in vitro study using AAA patient-derived SMCs [8], we observed significant differences in the expression of genes related to metabolic activity between non-diabetic (ND) and diabetic (D) patients. Additionally, we discovered that metformin enhances contractility and oxidoreductase activity while reducing proliferation, migration, and inflammation in aortic SMCs. In other studies, metformin protected the extracellular matrix (ECM) from degradation by preserving elastin fibers and decreasing matrix metalloproteinase (MMP) expression.

Impaired mitochondrial function may cause SMC phenotypic changes, exhaustion, increased reactive oxygen species (ROS) production and SMC apoptosis, all of which are observed in AAA [9]. ROS, byproducts of aerobic metabolism or inflammatory pathways, activate protective responses to manage oxidative stress and maintain redox balance. However, oxidative stress occurs when ROS production exceeds antioxidant capacity, which may contribute to AAA progression [10,11]. Redox stress is a type of cellular stress caused not only by oxidative molecules but also by other reactive species, such as reactive nitrogen species (RNS) and electrophiles. It has previously been shown that SMC mitochondrial dysfunction has a significant impact on ECM composition and vice versa, which is thus highly relevant during vascular disease [12].

To further explore the potential beneficial effects of long-term metformin use on AAA growth, we performed a proteomics analysis on SMCs derived from non-pathological aortic controls (C), ND-AAA patients and D-AAA patients treated with metformin. Key findings were subsequently validated in aortic tissue using mass spectrometry-based proteomics. To determine whether the observed differences were explicitly due to metformin use, we conducted in vitro experiments on patient-derived AAA SMCs to verify our proteomics results.

## 2. Methods

An extended version is described in the Supplementary Material section, and detailed information about the resources can be found in the Major Resources Table.

### 2.1. Patient Samples

#### 2.1.1. Patient Population

For this study, blood samples and aortic biopsies were obtained from patients who underwent open repair surgery for AAA between 2016 and 2024 at Amsterdam UMC or Dijklander Hospital, both located in The Netherlands. Before surgery, all patients provided informed consent for their blood and tissue to be stored in the Biobank for Aortic Aneurysms, Atherosclerosis, and Biomarkers (TcB: 2017.121). Control aortic biopsies were obtained from non-pathological aortas of postmortem heart-beating kidney donors, taken from the abdominal aorta at the level of the renal artery origin. SMC proteomics analysis was performed on smooth muscle cells cultured from a total of 32 aortic biopsies (controls *n* = 8; ND-AAA *n* = 19; D-AAA *n* = 5). Key findings were further validated in aortic tissue using proteomics on additional samples (controls *n* = 17; ND-AAA *n* = 42; D-AAA *n* = 15). No matching criteria were applied. The following patient characteristics were reported: age at the time of biopsy, sex, aneurysm size (mm), rupture, hypertension, previous vascular surgery, renal dysfunction and body mass index (BMI). Since the kidney donors remained anonymous, their only reported clinical characteristics were age and sex. For the qPCR experiments evaluating the effect of metformin, SMCs were obtained from six AAA patients included in the SMC proteomics analysis and nine additional AAA patients. For other experiments, there was no overlap between the AAA SMCs used and those included in the SMC proteomics analysis. For this study, blood samples from 12 ND-AAA patients and 10 D-AAA patients were obtained from the biobank and analyzed. All patient material was collected according to the Declaration of Helsinki regulations and the institutional guidelines of the Medical Ethical Committee of Amsterdam UMC, location VU Medical Center. Biobank material release was approved by the Biobank Review Committee of VUmc (TcB VUmc) under approval code 2017.121 [U2019.031].

#### 2.1.2. Aortic Biopsy and Cell Culture

Right after the surgeon removed the aortic tissue from the area of maximal dilation during the operation, the sample was immediately placed in NaCl solution and kept at 4 °C until it was transported on ice to the laboratory. A part of the intact aortic tissue was sectioned and placed in an aluminum cryo tube, flash-frozen in liquid nitrogen and stored at −80 °C until further processing. From the remaining aortic tissue, the intima and adventitia layers were removed, and the medial layer was cut into small explants and cultured in M231 (Medium231, Smooth Muscle Cell medium, Gibco, Life Technologies, Carlsbad, CA, USA) supplemented with Penicillin (100 U/mL) and Streptomycin (100 µg/mL) (Gibco, Life Technologies) and 5% Smooth Muscle Growth Supplement (SMGS, Gibco, Life Technologies), as described in the Supplemental Material. Primary SMCs were used between passages one and nine in all experiments.

#### 2.1.3. Plasma Samples

Before the incision, blood samples were collected in 6 mL EDTA tubes and transported to the laboratory at room temperature (RT). Samples were centrifuged at 2000× *g* for 10 min at RT, after which the plasma was collected and stored at −80 °C.

### 2.2. Proteomics Analysis

#### 2.2.1. SMC Proteomics Analysis

##### SMC Sample Preparation

A power calculation to determine the sample size for both aortic controls and AAA patients was performed and is described in our previous study [13]. However, no power calculation was done for the sample size of ND-AAA-SMC and D-AAA-SMC as two separate study groups within the whole cohort. Every SMC line was cultured in 15 cm dishes until 70–80% confluency was reached. After washing twice with cold phosphate buffered saline (PBS), cells were lysed using a lysis buffer and detached with a cell scraper. The process was completed within 1 min to preserve protein states. Protein concentration was measured with a BCA Protein Assay Kit (Thermo Fisher Scientific, Bremen, Germany). As a quality control, samples were loaded on a precast mini gel (Invitrogen, Waltham, MA, USA). Electrophoresis was conducted at 200 V in NuPAGE MES SDS running buffer until the dye reached the bottom of the gel. The gels were then fixed in ethanol–phosphoric acid solution and stained with 0.1% coomassie brilliant blue G-250 solution (Figure S1). Lysates were stored at −80 °C until further use. Lysates were thawed, and insoluble material was removed by centrifugation. Samples were reduced with dithiothreitol (4 mmol/L, 30 min at 55 °C) and alkylated with iodoacetamide (10 mmol/L, 15 min in the dark). The solution was then diluted to 2 mol/L urea by adding 20 mmol/L HEPES (pH 8.0) and digested overnight at RT with sequencing-grade modified trypsin (Promega, Madison, WI, USA) at a final concentration of 5 µg/mL. Digests were acidified with trifluoroacetic acid (TFA) to a final concentration of 0.1% and desalted using Oasis HLB cartridges (500 mg sorbent; Waters, Milford, MA, USA) equilibrated in 0.1% TFA. Bound peptides were washed twice with 0.1% TFA, eluted with 80% acetonitrile/0.1% TFA and lyophilized.

##### SMC Peptide Digestion and Mass Spectrometry

Peptide digests were dissolved in 20 µL of 0.5% TFA with 4% acetonitrile before injection; 5 µL was injected using partial loop injection. Peptides were separated using an Ultimate 3000 nano-LC-MS/MS system (Dionex LC-Packings, Amsterdam, The Netherlands) equipped with a 50-cm, 75-mm ID C18 Acclaim pepmap column (Thermo Scientific, Waltham, MA, USA). After injection, peptides were trapped at 3 mL/min on a 10 mm, 75 mm ID Acclaim Pepmap trap column (Thermo Scientific) in buffer A (0.1% formic acid) and separated at 300 mL/min with buffer B (80% acetonitrile/0.1% formic acid) gradient from 10% to 40% for 90 min (120 min inject to inject). Eluting peptides were ionized at +2 kV and introduced into a Q Exactive HF mass spectrometer (Thermo Fisher, Bremen, Germany). Intact masses were measured in the Orbitrap cell with a resolution of 120,000 (at *m*/*z* 200) using an automatic gain control target value of 3 × 10^6^ charges. The top 15 highest signal peptides (charge states ≥2+) were submitted to MS/MS in the higher energy collision cell (1.6-Da isolation width, 25% normalized collision energy). MS/MS spectra were measured in the Orbitrap with a resolution of 15,000 (at *m*/*z* 200) using an automatic gain control target value of 1 × 10^6^ charges and an underfill ratio of 0.1%. Dynamic exclusion was used with a repeat count of 1 and an exclusion time of 30 s.

##### SMC Protein Quantification

MS/MS spectra were searched against a Swissprot reference proteome (human, 2021_01 canonical plus isoforms, 42,383 entries) using MaxQuant 1.6.10.43. Enzyme specificity was set to trypsin, and up to two missed cleavages were allowed. Cysteine carboxamidomethylation (+57.021464 Da) was treated as a fixed modification and methionine oxidation (+15.994915 Da) and N-terminal acetylation (+42.010565 Da) as variable modifications. Peptide precursor ions were searched with a maximum mass deviation of 4.5 ppm and fragment ions with a maximum mass deviation of 20 ppm. Peptide, protein and site identifications were filtered at a false discovery rate of 1% using the decoy database strategy. The minimum peptide length was set at seven amino acids, the minimum Andromeda score for modified peptides was 40 and the corresponding minimum delta score was 6 (default MaxQuant settings).

##### SMC Proteomics Data Analysis

Protein-level differential analyses were performed using spectral count data. Counts were normalized to the total count per sample relative to the average sample total in the dataset. Group differences were tested using a β-binomial test for the independent samples [14]. In addition to a significant *p* value (*p* < 0.05), data were filtered to find the most discriminatory changes. Proteomics data were filtered for proteins found in ≥50% of the samples within one or both groups to compare C-SMC and ND-AAA-SMC. Proteomics data were filtered for proteins found in ≥75% of the samples within one or both groups to compare ND-AAA-SMC and D-AAA-SMC, to account for the smaller sample size. Venn diagrams were conducted using jvenn [15]. KEGG and Reactome pathway and Gene Ontology (GO) analyses were performed using ShinyGO 0.81 [16,17], with the complete set of identified proteins serving as the background for the KEGG and GO analyses. Results were filtered based on a False Discovery Rate (FDR) threshold, and only terms with at least two proteins were included. KEGG pathways were selected based on their relevance to the objectives of this study. Overlapping GO terms involving the same protein group were reduced by selecting a single representative term. Visualizations were generated using SR Plot [18]. Protein–protein interaction (PPI) analysis was performed using the STRING database (version 12.0) and visualized with Cytoscape software (version 3.10.3) [19,20]. PPI networks only contained proteins identified in our proteomics screen, no additional enrichment or expansion was applied. Hub proteins were identified using the CytoHubba program version 0.1, with the Maximal Clique Centrality (MCC) method [21]. Other Graphs were constructed using GraphPad Prism 10.2.0 (GraphPad Software, San Diego, CA, USA) and SR plot.

##### Data Availability

The mass spectrometry proteomics data have been deposited to the ProteomeXchange consortium via the PRIDE (Proteomics Identifications) [22] partner repository with the SMC dataset identifier PXD054353.

#### 2.2.2. Tissue Proteomics Analysis

To validate SMC proteomics findings in the intact aortic wall, protein expression of selected proteins from the identified PPI networks were analyzed in aortic tissue using a proteomics approach. Briefly, tissues were homogenized using zirconium oxide beads and lysed in RIPA buffer with protease and phosphatase inhibitors. For each sample, 10 µg of total protein was processed by S-Trap suspension trapping [23,24] for clean-up and trypsin digestion after reduction and alkylation. For each digest, 600 ng peptides were loaded on an Evotip and were separated on an Evosep One (Evosep, Odense, Denmark) nanoLC equipped with a Pepsep C18 column at 30 SPD. Eluting peptides were ionized in a captive spray device at 1400V and measured in a timsToF HT mass spectrometer (Bruker, Bremen, Germany). Data were acquired in DIA-PASEF mode and were searched against a predicted human spectral library using DIA-NN v1.9.2 [25]. Protein group intensities were quantified by DIA MaxLFQ [26]. Further tissue proteomics details can be found in Supplementary Materials. The mass spectrometry proteomics data have been deposited to the ProteomeXchange consortium via the PRIDE (Proteomics Identifications) [22] partner repository with the tissue dataset identifier PXD067859.

### 2.3. Experiments to Test the Effect of Metformin on SMCs

#### 2.3.1. Metformin Dosage

The concentration of 10 mM metformin hydrochloride (Toronto Research Chemicals Inc., Toronto, ON, Canada) was chosen based on the literature and the preliminary results of our previous study, in which we determined that this concentration is not harmful to cells in short-term cell culture experiments [8].

#### 2.3.2. RNA Isolation and Quantitative Polymerase Chain Reaction

To test the effect of metformin on cytoskeleton, ECM, mitochondrial markers and genes involved in the reaction against redox stress in SMCs, mRNA expression levels of specific genes, were measured. Real-time quantitative Polymerase Chain Reaction (RT-qPCR) was used to assess the expression of 16 genes (primer sequences are provided in Table S1). The mRNA levels of target genes were normalized to the housekeeping gene TATA Box Binding Protein (*TBP*). Gene expression analysis used the 2^−ΔCT^ method.

#### 2.3.3. Western Blotting

SMCs were washed with PBS and lysed in 130 µL SDS sample buffer. The protein concentration was measured with a Pierce BCA Protein Assay Kit (Thermo Fisher Scientific, Waltham, MA, USA), according to the manufacturer’s instructions, to ensure equal protein loading across samples. After boiling the lysates at 95 °C for 10 min, 15 µL of each sample was loaded onto SDS-PAGE gels for protein separation. Proteins were transferred to nitrocellulose membranes, blocked with 5% bovine serum albumin (BSA) for 1 h at RT. Primary antibodies against Phospho-AMPKα (Thr172) (Cell Signaling Technology, Danvers, MA, USA, 1:1000), AMPKα (Cell Signaling Technology, 1:1000), KEAP1 (Proteintech (Proteintech Group Inc., Rosemont, IL, USA) 1:1500) and β-Actin (Cell Signaling Technology, 1:1000) as loading control were incubated overnight at 4 °C. Secondary antibody incubation was performed for 1 h at RT with HRP conjugated polyclonal goat anti-rabbit immunoglobulin (Dako (Carpinteria, CA, USA), 1:5000), diluted in milk powder. Proteins were visualized with enhanced chemiluminescence (Amersham/GE Healthcare, Little Chalfont, UK) using an Amersham Imager 600 (GE Healthcare). The band intensities were analyzed using ImageQuant TL (GE Healthcare).

#### 2.3.4. Immunofluorescence

The SMCs were cultured in 96-well cell culture microplates with F-bottom (Greiner Bio-One, Alphen aan den Rijn, South Holland, The Netherlands). Cells were fixed and stained for NAD(P)H Quinone Dehydrogenase 1 (NQO1) after four days of metformin treatment and for Nuclear Factor Erythroid 2-Related Factor 2 (NRF2) after three days. Cytoskeletal F-actin was stained with Acti-Stain 670 Phalloidin (Cytoskeleton Inc. (Denver, CO, USA), 1:250), nuclei were stained with DAPI (4′,6-diamidino-2-phenylindole) (Thermo Fisher Scientific, 1:1000), and specific antibodies for anti-phospho-Nrf2 Ser40 (Abcam, 1:250) and NQO1 (Cell signaling Technology, 1:150; Danvers, MA, USA) were used for staining. Images were captured using an ImageXpress Pico Automated Cell Imaging System Micro 4 (Molecular Devices, San Jose, CA, USA) and were analyzed using the associated software CellReporterXpress version 2.9. Confocal z-stacked images were made using the 60× objective on the spinning disk microscope (Nikon Eclipse Ti2, Nikon Corporation, Tokyo, Japan) and visualized using ImageJ 1.49 (National Institutes of Health, Bethesda, MD, USA).

#### 2.3.5. Pentosidine Measurement

The ELISA kit for Pentosidine (CLOUD-CLONE CORP, Houston, TX, USA) was used according to the manufacturer’s protocol to measure the advanced glycation end products (AGEs) generation in cell culture supernatants.

#### 2.3.6. Baseline Measurement of Oxygen Consumption and Extracellular Acidification

Mitochondrial oxygen consumption rate (OCR) and extracellular acidification rate (ECAR) were measured using the Seahorse XFe96 Analyzer (Agilent, Santa Clara, CA, USA), as described in the Supplementary Material section. The values were normalized by counting cell nuclei using Hoechst 33342 (Thermo Fisher Scientific, 1:1000).

#### 2.3.7. L-Lactate Assay

An L-Lactate assay (Roche, Basel, Switzerland) was performed on cell culture supernatants to determine the effect of metformin on anaerobic glycolysis.

#### 2.3.8. NQO1 Activity Assay Kit

The NQO1 Activity Assay kit (Abcam, Cambridge, UK) was used according to the manufacturer’s protocol to determine the NQO1 enzymatic activity as proxy for the amount of NQO1 protein levels in plasma.

#### 2.3.9. Statistics

Statistical analyses were conducted using IBM SPSS Statistics (version 28, IBM Corp., Armonk, NY, USA). Both ANOVA and χ^2^ tests were used to evaluate clinical characteristics across three study groups. The independent samples *t*-test and χ^2^ test were utilized to compare patient characteristics between two study groups. The statistical analysis of patient characteristics did not account for missing data. When datasets were normally distributed, either the paired *t*-test or the independent samples *t*-test was used. A two-sided *p*-value is reported, except when a one-sided test was applied due to a specific directional hypothesis, and this is explicitly stated in the text. When datasets were not normally distributed, nonparametric tests were applied. The Mann–Whitney U test was used to compare two independent groups. The Wilcoxon Signed-Ranks Test was performed to assess differences between related groups (the untreated and metformin-treated groups). Results are shown in plots with the mean and standard deviation (SD) for parametric tests, while plots representing the median and interquartile range (IQR) are used for nonparametric tests. Statistical significance was defined as *p* < 0.05. Plots were generated using GraphPad Prism version 10.2.0.

## 3. Results

### 3.1. Clinical Characteristics

Clinical characteristics of samples included in the SMC proteomics analysis are summarized in Table 1. Comparing age at the time of biopsy between the three study groups, a significant difference was observed (*p* < 0.001), where ND-AAA and D-AAA patients were older than the controls (72.1 ± 9.1, 75.0 ± 7.8 and 52.4 ± 16.7 respectively). The distribution by sex was equal across all study groups. Comparing the ND-AAA and D-AAA patients, there was no significant difference in age, sex, aneurysm size, rupture incidence, smoking status, previous vascular surgery or BMI. However, the prevalence of hypertension and renal dysfunction was higher in D-AAA patients, as compared to ND-AAA (100.0% vs. 43.8%, *p* = 0.045; and 80.0% vs. 6.3%, *p* = 0.004, respectively).

Clinical characteristics of samples included in the aortic tissue proteomics analysis are summarized in Table S2. Age at the time of biopsy and sex distribution differed significantly between the three groups (*p* < 0.001 and *p* = 0.013, respectively), with controls being younger and more often female than ND-AAA and D-AAA patients. No significant differences were observed between ND-AAA and D-AAA patients in age, sex, aneurysm size, smoking status, prevalence of hypertension or renal dysfunction, or BMI. However, previous vascular surgery was significantly more common in D-AAA patients than in ND-AAA patients (66.7% vs. 29.3%, *p* = 0.015).

### 3.2. Differences in Proteomic Profile Between SMCs from Controls, Non-Diabetic and Diabetic AAA Patients

**Figure 1:**
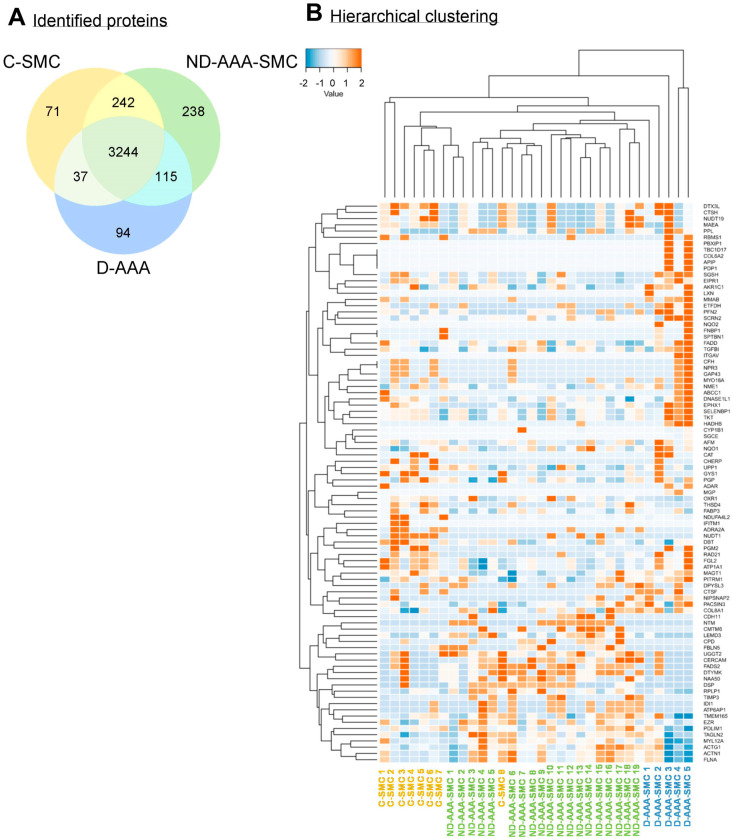
**Differences in the proteome of aortic SMC derived from controls, non-diabetic and diabetic AAA patients.** (**A**) Venn diagram of identified proteins in cultured aortic SMCs derived from controls (C-SMC) (*n* = 8, orange), non-diabetic AAA patients (ND-AAA-SMC) (*n* = 19, green) and diabetic AAA patients (D-AAA-SMC) (*n* = 5, blue) and the overlap between the groups. (**B**) Hierarchical clustering of the 90 proteins that were significant differentially expressed (*p* < 0.05) between C-SMC (yellow), ND-AAA-SMC (green) and D-AAA-SMC (blue). The proteomics spectral count data were tested using a β-binomial test for independent samples [14].

In the proteomics screen of aortic SMCs from controls, ND-AAA and D-AAA patients, 4523 proteins were identified (Figure 1A) (Table S3), and comparison of the three groups revealed 90 significantly differentially expressed proteins (Table S4). The hierarchical clustering analysis of these 90 differential proteins demonstrated that the study groups cluster based on their protein profiles (Figure 1B). Remarkably, SMCs isolated from D-AAA patients exhibit a distinct protein expression pattern compared to the other groups, suggesting persistent epigenetic changes in culture, potentially induced by long-term metformin use or the diabetic condition itself.

### 3.3. Differences in Proteomic Profile Between SMCs from Non-Diabetic AAA Patients and Aortic Controls

**Figure 2:**
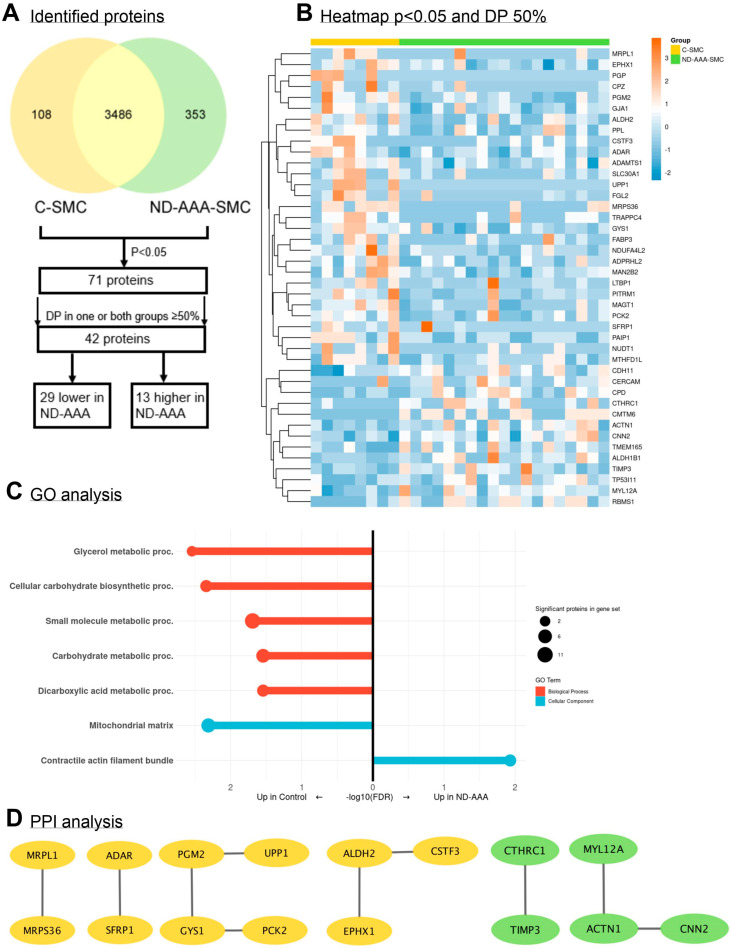
**Differences in the proteome of aortic SMC derived from controls and non-diabetic AAA patients.** (**A**) Number of identified proteins found in C-SMCs (yellow) and ND-AAA-SMCs (green) filtered for significance (*p* < 0.05). Proteins were further filtered for ≥50% data presence (DP) in one or both groups, resulting in 42 proteins, of which 29 were less expressed in ND-AAA-SMCs and 13 were more expressed in ND-AAA-SMCs. The proteomics spectral count data were tested using a β-binomial test for independent samples [14]. (**B**) Heatmap of the proteins, after filtering for significance (*p* < 0.05) and DP ≥ 50% in one or both groups, clustered on protein expression. (**C**) Results of the GO annotation analysis for the 29 proteins with reduced expression in ND-AAA-SMCs (shown on the left) and the 13 proteins with elevated expression in ND-AAA-SMCs (shown on the right), compared to C-SMCs. GO terms are categorized by Biological Process (BP, in red) and Cellular Component (CC, in blue). Proc. = process. Identifiers of the GO terms can be found in Table S6. (**D**) Protein–protein interaction (PPI) network analyses performed separately for the increased and decreased proteins in ND-AAA-SMC compared to C-SMC; proteins with lower expression in ND-AAA-SMCs highlighted in yellow, and proteins with higher expression compared to C-SMCs highlighted in green.

Proteomic differences were analyzed to assess functional alterations in SMCs cultured from aortic tissue from ND-AAA patients compared to controls. Comparison of the proteomic profiles revealed 71 proteins with significantly different expression levels between the two groups. After applying a ≥50% data presence filter in one or both groups, the list was reduced to 42 proteins. Of these, 29 were less expressed in ND-AAA-SMCs, while 13 were more expressed compared to C-SMCs (Figure 2A,B; Table S5).

The 29 proteins that showed reduced abundance in ND-AAA-SMCs were further investigated using KEGG and Reactome pathway enrichment analysis and gene ontology (GO) annotation analysis (Figure 2C). The GO annotation revealed alterations in metabolic processes and in the mitochondrial matrix. No significant enrichment was observed for biological pathways (KEGG) and Reactome pathways or molecular functions. For the 13 proteins that showed enhanced abundance in ND-AAA-SMCs, GO annotation analysis identified the cellular component GO term ‘contractile actin filament bundle’. No significant enrichment was observed for KEGG and Reactome pathways, biological processes or molecular functions.

PPI analysis was performed separately for the 29 proteins with decreased abundance and the 13 proteins with increased abundance in ND-AAA-SMCs compared to C-SMCs (Figure 2D), as well as for all proteins combined (Figure S2). Among the 29 proteins with reduced abundance, 11 were connected to at least one other protein. Aldehyde Dehydrogenase 2 Family Member (ALDH2) and Epoxide Hydrolase 1 (EPHX1) neutralize reactive metabolites and ROS under oxidative stress conditions [27,28]. Glycogen Synthase 1 (GYS1) is responsible for synthesis of glycogen from glucose [13]; Phosphoglucomutase 2 (PGM2) is involved in the conversion of glucose-1-phosphate to glucose-6-phosphate, a critical step in glycogen metabolism and Phosphoenolpyruvate Carboxykinase 2 (PCK2) negatively regulates mitochondrial respiration and is involved in the gluconeogenesis pathway. Reduced expression of these proteins in ND-AAA-SMCs suggest more glucose consumption. Interestingly, ALDH2 deficiency is associated with the development of AAAs in humans and a murine model. ALDH2 protects against AAA formation by reducing ROS, vascular inflammation and SMC apoptosis [27].

Five of the 13 proteins with elevated abundance were connected to at least one other protein. Myosin Light Chain 12A (MYL12A), Actinin Alpha 1 (ACTN1) and Calponin 2 (CNN2) all play a role in the SMC contractility and cytoskeleton organization. Tissue Inhibitor of Metalloproteinase 3 (TIMP3) inhibits the catalytic activity of MMPs and can promote SMC apoptosis [29]. Interestingly, TIMP3 is highly overexpressed in AAA tissue [30], and deficiency of TIMP3 in an aneurysm mouse model showed enhanced AAA development [31]. Collagen Triple Helix Repeat Containing 1 (CTHRC1) is known to be rapidly expressed following arterial injury and plays a role in the fibrotic wound-healing process [32].

These findings suggest that metabolic processes and mitochondrial function are notably altered in ND-AAA-SMCs, as previously reported in AAA tissue [33,34]. The elevated proteins in ND-AAA-SMCs primarily correspond to cytoskeleton and ECM changes, part of the SMC mechanosensitive machinery. Interestingly, almost all genes involved in hereditary aneurysm development are part of this mechanosensing pathway [35,36]. Thus, the cultured AAA-SMCs seem to represent aortic aneurysm pathology.

### 3.4. Differences in Proteomic Profile Between SMCs from Diabetic and Non-Diabetic AAA Patients

To uncover mechanisms underlying the inverse association of diabetes, which can potentially be related to metformin use, and AAA growth [3,6], we compared the proteomic profiles of SMCs from D-AAA patients (all using metformin) with ND-AAA patients.

**Figure 3:**
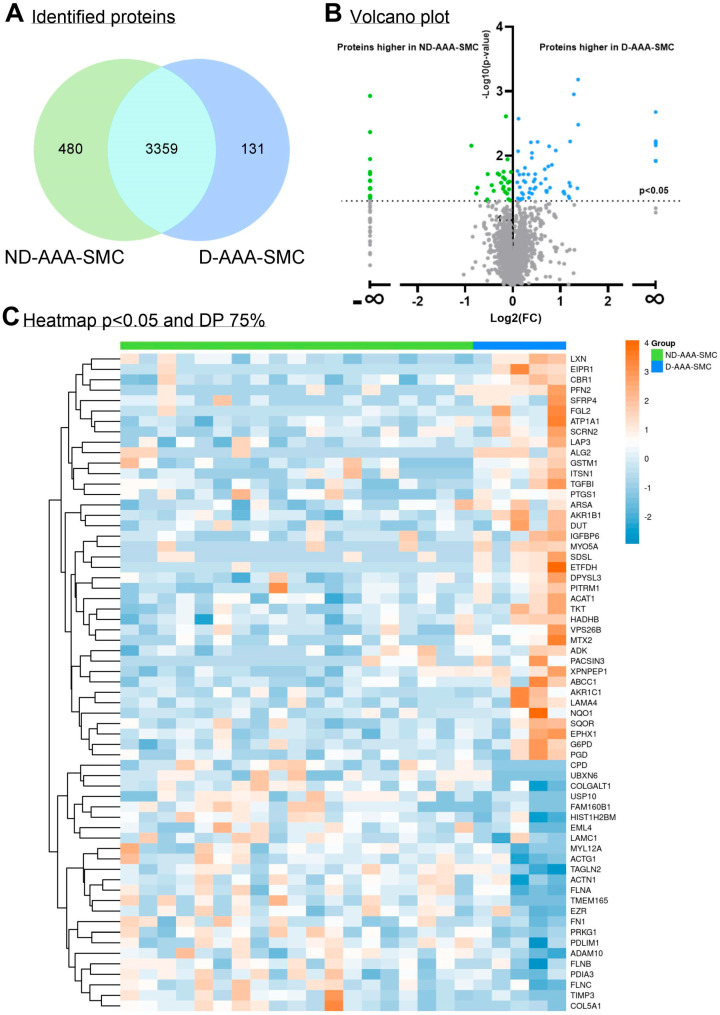
**Differences in the proteome of aortic SMC derived from non-diabetic and diabetic AAA patients.** (**A**) Venn diagram of identified proteins in non-diabetic AAA SMCs (ND-AAA-SMC) (*n* = 19, green) and diabetic AAA SMCs (D-AAA-SMC) (*n* = 5, blue) and the overlap between the groups. (**B**) Volcano plot illustrating proteins significantly differentially expressed between ND-AAA-SMCs and D-AAA-SMCs. A negative Log2 (fold change (FC)) indicates higher expression of the protein in ND-AAA-SMCs compared to D-AAA-SMCs, while a positive Log2(FC) indicates higher expression of the protein in D-AAA-SMCs compared to ND-AAA-SMCs. An Log2(FC) of −∞ or ∞ indicates that the protein was uniquely present in either the ND-AAA-SMC group or the D-AAA-SMC group, respectively. (**C**) Heatmap of the 63 proteins, after filtering for significance (*p* < 0.05) and DP ≥ 75% in one or both groups, clustered on protein expression. The proteomics spectral count data were tested using a β-binomial test for independent samples [14].

The Venn diagram in Figure 3A depicts the number of identified proteins in D-AAA-SMC and ND-AAA-SMC. A comparison of the proteomic profile of D-AAA-SMC and ND-AAA-SMC revealed 115 proteins with significant differences in expression (Figure 3B), combining with an ≥75% data presence filter in one or both groups resulted in 63 proteins (Figure 3C; Table S7); 24 proteins were less expressed in D-AAA-SMC compared to ND-AAA-SMC, while 39 were more expressed in D-AAA-SMC compared to ND-AAA-SMC.

**Figure 4:**
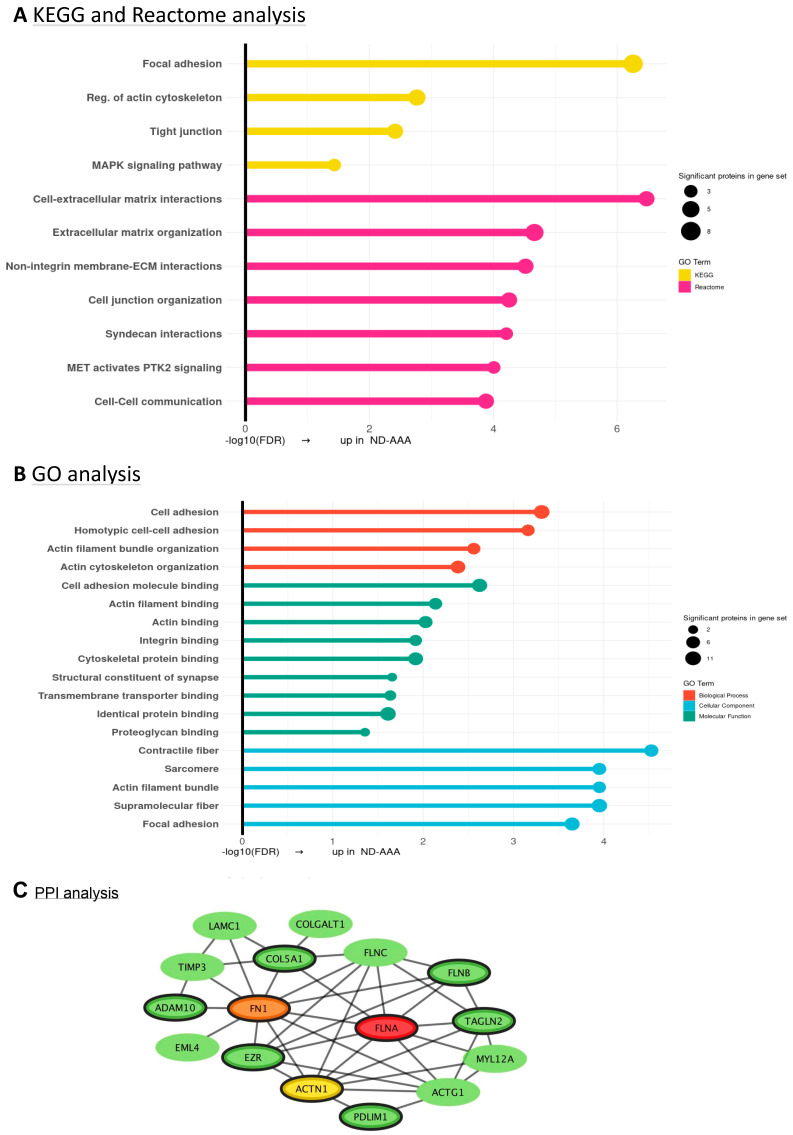
**Analysis of proteins with decreased expression in aortic SMC derived from diabetic AAA patients compared to non-diabetic AAA patients.** (**A**) Results of the KEGG (yellow) and Reactome (pink) pathway enrichment analysis of the proteins with decreased expression in D-AAA-SMCs compared to ND-AAA-SMCs, after filtering for significance (*p* < 0.05) and DP ≥ 75% in one or both groups. Identifiers of the KEGG and Reactome terms can be found in Table S6. (**B**) Results of the GO annotation analysis of the 24 proteins with reduced expression in D-AAA-SMCs compared to ND-AAA-SMCs. GO terms are categorized by Biological Process (BP, in red), Molecular Function (MF, in green) and Cellular Component (CC, in blue). Identifiers of the GO terms can be found in Table S6. (**C**) Results of the protein–protein interaction (PPI) analysis of the 24 proteins with decreased expression in D-AAA-SMCs compared to ND-AAA-SMCs (green). Overall, 16 of the 24 proteins are part of the PPI network, with Filamin A (FLNA, red), Fibronectin 1 (FN1, orange) and Actinin Alpha 1 (ACTN1, yellow) identified as the top three hub proteins. Within this network, nine proteins, including the hub proteins, were associated with the molecular function GO term ‘cell adhesion molecule binding’ and are highlighted with a thick outline.

The 24 less expressed proteins in D-AAA-SMCs were further investigated using KEGG and Reactome pathway enrichment analysis, GO annotation analysis and protein–protein interaction (PPI) network analysis. The KEGG and Reactome pathway enrichment results and the GO annotation findings include mostly cytoskeletal and cell adhesion changes (Figure 4A,B). PPI analysis revealed that 16 of the 24 proteins are part of the PPI network, with Filamin A (FLNA), Fibronectin 1 (FN1) and Actinin Alpha 1 (ACTN1) identified as the top three hub proteins (Figure 4C). The expression of the hub proteins was lower in D-AAA-SMCs and C-SMCs compared to ND-AAA-SMCs, suggesting a potential normalization effect in D-AAA patients treated with metformin. However, among these comparisons, only the difference in ACTN1 expression between ND-AAA-SMCs and C-SMCs reached statistical significance (Figure S4A). FLNA stabilizes the cytoskeleton and plays a crucial role in signal transduction. Its overexpression promotes SMC proliferation and migration [37]. Moreover, mutations in FLNA are known to cause aortic aneurysms [38,39]. ACTN1 is another cytoskeletal protein that stabilizes actin filaments, supports cell adhesion and migration, and maintains mechanical stability. Other cytoskeletal proteins in this network are Ezrin (EZR), Echinoderm Microtubule-Associated Protein-Like 4 (EML4), PDZ and LIM Domain Protein 1 (PDLIM1), Transgelin-2 (TAGLN2), Myosin Light Chain 12A (MYL12A), and Filamin B and C (FLNB and FLNC), along with Actin Gamma-1 (ACTG1). FN1 is an ECM protein secreted by proliferative smooth muscle cells during vascular repair and pathological remodeling processes, accumulating in the medial layer of the aorta [40]. Moreover, it is involved in collagen and Fibrillin-1 (FBN1) fiber formation, where mutations in collagens or FBN1 are known to give aortic aneurysms [38,39]. Other proteins in this network associated with ECM include TIMP3, A Disintegrin and Metalloproteinase 10 (ADAM10), Collagen Type V Alpha-1 (COL5A1), Collagen Beta(1-O)Galactosyltransferase 1 (COLGALT1) and Laminin Subunit Gamma-1 (LAMC1), of which ADAM10, COL5A1 and TIMP3 have been associated with aneurysm formation [30,31,41,42]. Within this network, nine proteins including the hub proteins, were associated with the molecular function GO term ‘cell adhesion molecule binding’ and are highlighted with a thick outline.

**Figure 5:**
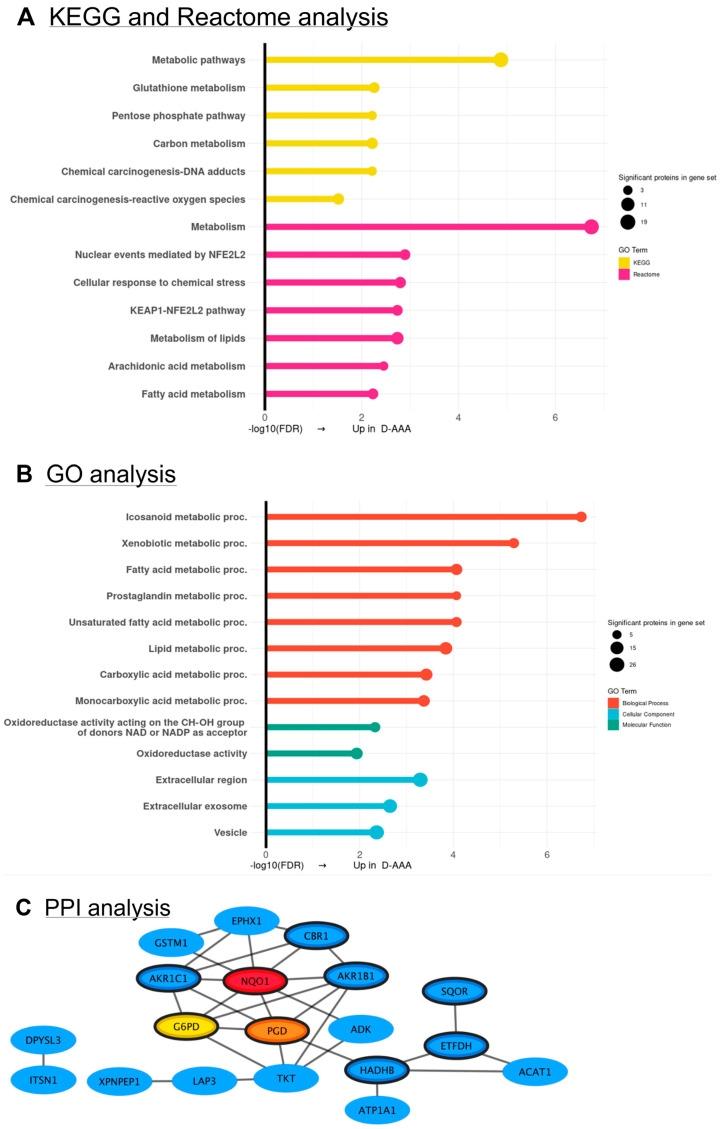
**Analysis of proteins with increased expression in aortic SMC derived from diabetic AAA patients compared to non-diabetic AAA patients.** (**A**) Results of the KEGG (yellow) and Reactome (pink) pathway enrichment analysis of the 39 proteins with increased expression in D-AAA-SMCs compared to ND-AAA-SMCs, after filtering for significance (*p* < 0.05) and DP ≥ 75% in one or both groups. Identifiers of the KEGG and Reactome terms can be found in Table S6. (**B**) Results of the GO annotation analysis of the 39 proteins with increased expression in D-AAA-SMCs compared to ND-AAA-SMCs. GO terms are categorized by Biological Process (BP, in red), Molecular Function (MF, in green) and Cellular Component (CC, in blue). Proc. = process. Identifiers of the GO terms can be found in Table S6. (**C**) Results of the protein–protein interaction (PPI) analysis of the 39 proteins with increased expression in D-AAA-SMCs compared to ND-AAA-SMCs (blue). Nineteen proteins are part of the network, with the following three hub proteins: NAD(P)H Quinone Dehydrogenase 1 (NQO1, red), 6-Phosphogluconate Dehydrogenase (PGD, orange) and Glucose-6-Phosphate Dehydrogenase (G6PD, yellow). Within this network, nine proteins, including all the hub proteins, were associated with the molecular function GO term ‘oxidoreductase’ and are highlighted with a thick outline.

The results of the KEGG and Reactome pathway enrichment, GO annotation and PPI analysis on the 39 proteins that were elevated in D-AAA-SMCs compared to ND-AAA-SMCs are displayed in Figure 5. PPI analysis revealed that 19 of the 39 proteins are part of a network, with NAD(P)H Quinone Oxidoreductase 1 (NQO1), Glucose-6-Phosphate Dehydrogenase (G6PD) and 6-Phosphogluconate Dehydrogenase (PGD) identified as the top three hub proteins (Figure 5C). The expression of the hub proteins was higher in D-AAA-SMCs compared to ND-AAA-SMCs and controls; however, the difference relative to controls was not statistically significant (Figure S4B). NQO1 prevents ROS formation by catalyzing the detoxification of toxic arylating and oxidative quinones. Its antioxidant function is linked to its role in keeping lipid-soluble antioxidants in their reduced form, thereby protecting the cell membranes against lipid peroxidation [43]. G6PD and PGD are both primary pentose phosphate pathway (PPP) cycle enzymes and responsible for NAD(P)H generation that helps reduce oxidative stress by supporting the function of antioxidants, such as Aldo-Keto Reductases (AKR), NQO1, Glutathion-S-transferase M1 (GSTM1), Carbonyl Reductase 1 (CBR1) and glutathione reductase, which rely on NAD(P)H as a cofactor [44]. Another PPP enzyme in the network is transketolase (TKT). Within this network, nine proteins, including all three hub proteins were associated with the molecular function GO term ‘oxidoreductase’ and are highlighted with a thick outline. Additionally, the Reactome pathways ‘Nuclear events mediated by NFE2L2’ and ‘KEAP1-NFE2L2 pathway’ were significantly enriched. Notably, NFE2L2, also known as Nuclear Factor Erythroid 2-Related Factor 2 (NRF2), is a key transcription factor regulating resistance to oxidative stress and controlling the response to environmental stressors [44,45]. Together with the three hub proteins, CBR1, AKR1B1, AKR1C1, EPHX1, GSTM1 and TKT are all target genes of NRF2.

Interestingly, when testing all 63 proteins together for PPI analysis, the mechanosensing and antioxidant pathways are connected via regulation of the pentose phosphate pathway (Figure S3). Of note, the pentose phosphate pathway is a metabolic redox sensor and an ancient mechanism, going back to yeast [46]. This pathway branches off from glycolysis and plays a key role in balancing cellular energy and redox status by generating NAD(P)H to replenish low glutathione levels against oxidative damage. Moreover, NRF2 is likely involved, since it can regulate the pentose phosphate pathway and the expression of target genes involved in the antioxidant reaction [47]. Clearly, the antioxidant pathway is induced in the D-AAA-SMCs. A number of additional proteins have become part of the combined cluster, including two aneurysm-related proteins, Transforming Growth Factor Beta-Induced Protein (TGFBI) and Protein Kinase cGMP-Dependent 1 (PRKG1) [39].

### 3.5. Validation of SMC Proteomics Findings in Aortic Tissue

To explore whether the SMC proteomics findings are reflected in the intact aortic wall, protein expression of selected proteins from the identified PPI networks were analyzed in aortic tissue using a proteomics approach. Aortic tissue from controls (*n* = 17), ND-AAA (*n* = 42), and D-AAA (*n* = 15) was examined, resulting in the identification of 9422 proteins (Table S8).

**Figure 6:**
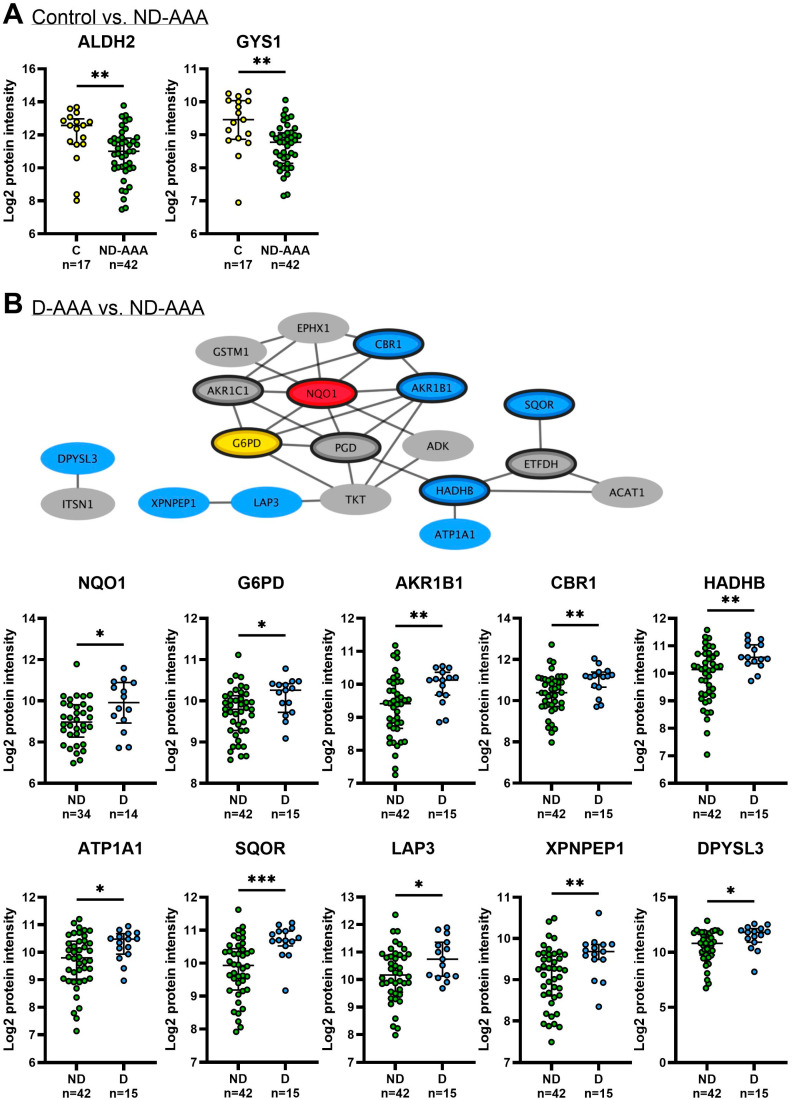
**Validation of SMC proteomics findings in aortic tissue.** (**A**) Log2 protein intensity in aortic tissue from control and non-diabetic AAA (ND-AAA) patients for ALDH2 (*p* < 0.009) and GYS1 (*p* = 0.001). (**B**) Previously reported protein–protein interaction (PPI) network of proteins with increased expression in diabetic AAA (D-AAA) SMCs compared to non-diabetic AAA (ND-AAA) SMCs. Proteins validated in aortic tissue are shown in color (*n* = 10), whereas non-validated proteins are depicted in grey (*n* = 9). Separate graphs of aortic protein expression are shown in Log2 protein intensity for the validated proteins; NQO1 (*p* = 0.033), G6PD (*p* = 0.021), AKR1B1 (*p* = 0.005), CBR1 (*p* = 0.007), HADHB (*p* = 0.005), ATP1A1 (*p* = 0.025), SQOR (*p* < 0.001), LAP3 (*p* = 0.035), XPNPEP1 (*p* = 0.007) and DPYSL3 (*p* = 0.016). Median with interquartile range is shown. Differences in protein abundances were tested using the Mann–Whitney U test for two-group comparisons, based on Log2-transformed intensity values. Green: expression in ND-AAA aortic tissue samples; Blue: expression in D-AAA aortic tissue samples. * *p* ≤ 0.050; ** *p* ≤ 0.010, *** *p* ≤ 0.001.

In the comparison between the controls and ND-AAA patients, the metabolic enzymes ALDH2 and GYS1 showed expression patterns consistent with the SMC proteomics findings (Figure 6A).

In the PPI network of proteins with increased expression in D-AAA-SMC compared to ND-AAA-SMC, the majority of the proteins, many of which are involved in metabolism, were confirmed in the aortic tissues (Figure 6B).

### 3.6. The Effect of Metformin on SMCs in Vitro

To determine whether long-term metformin use in D-AAA patients explains the proteomic differences between D-AAA and ND-AAA SMCs and aortic tissues, we investigated the effect of metformin on SMC functions related to the proteins identified in the proteomic screen, using patient-derived SMCs in vitro.

#### 3.6.1. The Effect of Metformin on Cytoskeletal and Extracellular Matrix Markers

To investigate the effect of metformin on gene expression, SMCs derived from AAA patients were cultured with 10 mM metformin for five days prior to RNA isolation. Metformin did not significantly affect the gene expression of the hub proteins *FLNA* and *ACTN1*, which play a role in cytoskeletal stabilization (*p* = 0.972 and *p* = 0.245, respectively), or the hub protein *FN1*, which is involved in the ECM (*p* = 0.975) (Figure S5). However, several other proteins from our proteomics analysis did exhibit expression changes in response to metformin treatment. In line with the D-AAA-SMCs, metformin significantly decreased the gene expression of *MYL12A* (*p* = 0.015), a cytoskeletal marker that showed higher protein expression in ND-AAA-SMCs compared to C-SMCs and D-AAA-SMCs in the proteomics analysis (Figure S5A). Furthermore, MMP inhibitor *TIMP3*, an ECM-related protein that exhibited higher protein expression in ND-AAA-SMCs than C-SMCs and D-AAA-SMCs in the proteomics analysis, showed a significant reduction in gene expression following metformin treatment (*p* = 0.005). Given its key role in wound healing, the ECM protein Collagen Type I Alpha 1 (*COL1A1*) was tested and showed significantly reduced expression after metformin treatment (*p* = 0.013) [12] (Figure S5B).

Apart from ECM remodeling proteins, ECM stabilization by crosslinking the collagens may also be beneficial during AAA formation. Chronic hyperglycemia, a hallmark of diabetes, causes the generation of AGEs, which can non-enzymatically cross-link ECM proteins. This contributes to ECM stability and increased vascular stiffness [48]. To assess the impact of metformin on AGE production, we quantified pentosidine levels in the supernatant of SMC cultures after three days of 10 mM metformin exposure. Metformin treatment significantly increased pentosidine levels (*p* = 0.050), suggestive of enhanced ECM stabilization (Figure S5B).

#### he Effect of Metformin on Mitochondria and the Oxidative Stress Defense Mechanism

**Figure 7:**
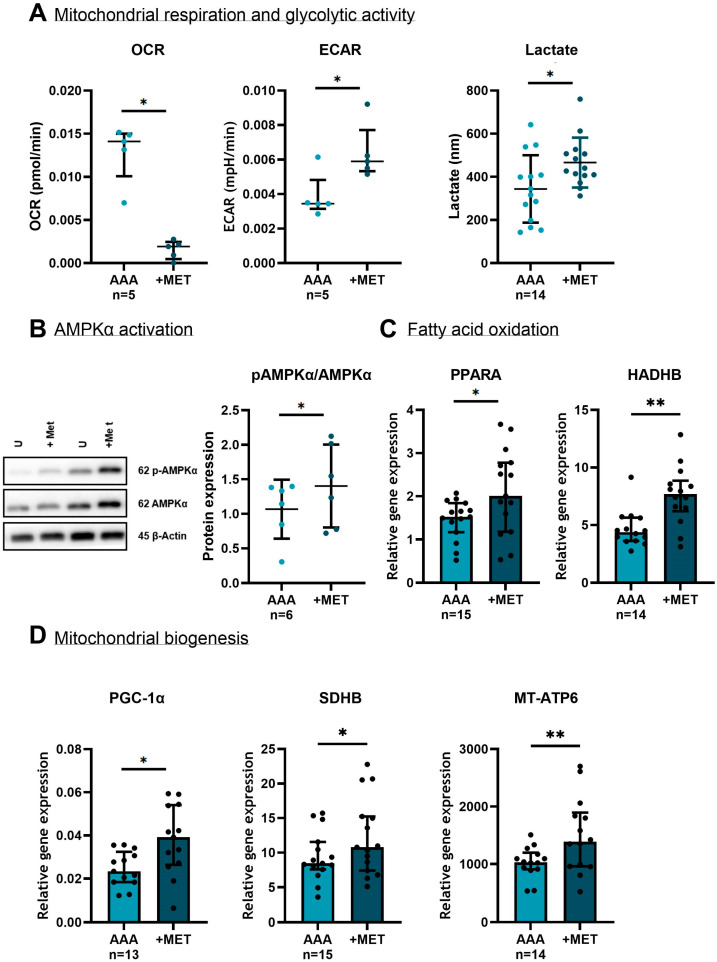
**The effect of metformin on mitochondrial respiration, glycolysis activity and mitochondrial biogenesis in aortic SMC derived from AAA patients.** (**A**) Metformin decreased the oxygen consumption rate (OCR) and increased the extracellular acidification rate (ECAR) (*p* = 0.043 for both). In line with the enhanced ECAR, the lactate levels in supernatant increased after treatment with metformin (one-sided *p* = 0.024). (**B**) To measure the AMP-Activated Protein Kinase Alfa (AMPKα) activity, the influence of metformin on the pAMPKα/AMPKα ratio was determined. There was a trend toward an increase in the pAMPKα/AMPKα ratio after metformin treatment (one-sided *p* = 0.039). β-Actin is shown as a loading control. Full unedited western blots can be found in Figure S6. (**C**) Gene expressions of Peroxisome Proliferator-Activated Receptor Alpha (*PPARA*) and Hydroxyacyl-CoA Dehydrogenase Trifunctional Multienzyme Complex Subunit Beta (*HADHB*) were elevated after metformin treatment (*p* = 0.011 and *p* = 0.004, respectively). (**D**) Metformin increased the gene expressions of Peroxisome Proliferator-Activated Receptor Gamma Coactivator 1-Alpha (*PGC-1α*) (*p* = 0.011), Succinate Dehydrogenase Complex Subunit B (*SDHB*) (*p* = 0.031) and Mitochondrially Encoded ATP Synthase Membrane Subunit 6 (*MT-ATP6*) (*p* = 0.008). The mRNA levels of target genes were normalized to the housekeeping gene TATA Box Binding Protein (*TBP*). The paired *t*-test was used when data were normally distributed (lactate and pAMPKα/AMPKα graphs), and the Wilcoxon Signed-Ranks Test was performed when data were not normally distributed (all other markers) to assess differences between the untreated and metformin treated SMCs. Data represent mean with standard deviation in the lactate and pAMPKα/AMPKα graphs and in all other graphs data represent the median and interquartile range. Light blue: expression in AAA samples; dark blue: expression in AAA samples +MET. * *p* ≤ 0.050; ** *p* ≤ 0.010. MET = metformin.

Mitochondrial respiration and glycolytic state were evaluated upon 10 mM metformin treatment for 24 h. The oxygen consumption rate (OCR) was significantly decreased, and the extracellular acidification rate (ECAR) was significantly increased after treatment with metformin (*p* = 0.043 for both). In line with ECAR, the lactate levels were higher in the supernatant after three days of 10 mM metformin treatment (one-sided *p* = 0.024) (Figure 7A).

AMP-activated protein kinase (AMPK) is a key energy sensor that promotes a metabolic shift toward energy-generating processes, such as increased fatty acid oxidation and mitochondrial biogenesis, in order to improve cellular energy production and restore energy balance. After 10 mM metformin treatment for six hours, there was an increase in phosphorylated-AMPK (pAMPKα)/AMPKα ratio, indicating an activation of the AMPK pathway (one-sided *p* = 0.039) (Figure 7B).

In addition, metformin caused increased gene expression of Peroxisome Proliferator-Activated Receptor Alpha (*PPARA*) and Hydroxyacyl-CoA Dehydrogenase Trifunctional Multienzyme Complex Subunit Beta (*HADHB*) (*p* = 0.011 and *p* = 0.004, respectively) (Figure 7C). Notably, the protein expression of HADHB was also higher in SMCs from D-AAA patients using metformin compared to ND-AAA patients, as shown by the proteomics analysis. PPARA is a nuclear receptor that regulates the expression of genes involved in mitochondrial and peroxisomal β-oxidation of fatty acids [12]. It is activated during fasting or energy stress when fatty acids become the primary energy source. HADHB is a subunit of the mitochondrial trifunctional protein (MTP) complex, which is involved in β-oxidation of long-chain fatty acids. These findings indicate that metformin modulates cellular energy metabolism by promoting fatty acid β-oxidation.

To assess mitochondrial biogenesis, the gene expression of Peroxisome Proliferator-Activated Receptor Gamma Coactivator 1-Alpha (*PGC-1α*), Succinate Dehydrogenase Complex Subunit B (*SDHB*) and Mitochondrially Encoded ATP Synthase Membrane Subunit 6 (*MT-ATP6*) was measured following metformin treatment. PGC-1α is a driver of mitochondrial biogenesis [49], while SDHB and MT-ATP6 are mitochondrial genes involved in complex II and V, respectively [12]. Increased gene expression was seen of *PGC-1α* (*p* = 0.011), *SDHB* (*p* = 0.031) and *MT-ATP6* (*p* = 0.008) upon metformin treatment (Figure 7D), indicative for increased mitochondrial biogenesis.

**Figure 8:**
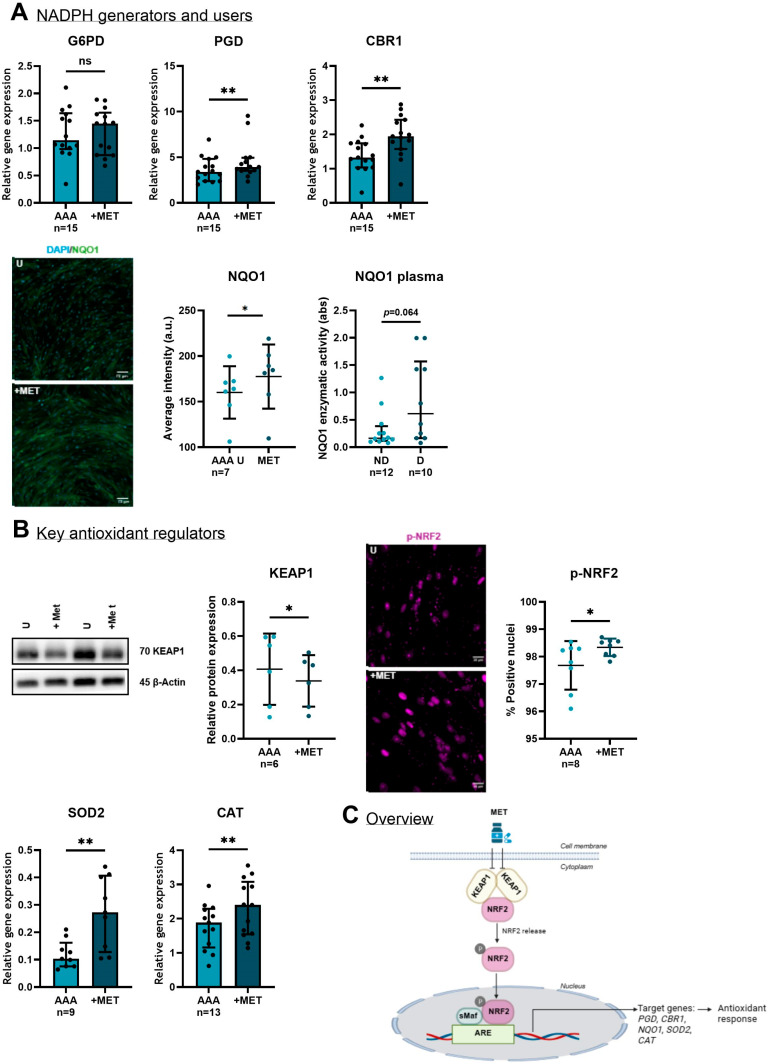
**The effect of metformin on NAPDH generators and users and key antioxidant regulators in aortic SMC derived from AAA patients.** (**A**) Although metformin did not significantly affect the gene expression of Glucose-6-Phosphate Dehydrogenase (*G6PD*) (*p* = 0.865), it increased the gene expression of Phosphogluconate Dehydrogenase (*PGD*) (*p* = 0.005), another NAD(P)H generator, and Carbonyl Reductase 1 (*CBR1*) (*p* = 0.009), an antioxidant enzyme that utilizes NAD(P)H as a cofactor. Additionally, metformin enhanced the protein expression of the antioxidant enzyme NAD(P)H Quinone Dehydrogenase 1 (NQO1), as demonstrated with representative immunofluorescence images (scale bar: 70 μM) and quantified using ImageXpress Pico Micro 4 software (*p* = 0.001). Furthermore, there was a trend toward higher NQO1 enzymatic activity in the plasma of D-AAA patients compared to ND-AAA patients (*p* = 0.064). (**B**) Metformin treatment decreased the protein expression of Kelch-Like ECH-Associated Protein 1 (KEAP1) (*p* = 0.047), a repressor of Nuclear Factor Erythroid 2-Related Factor 2 (NRF2), and increased the percentage of positive nuclei with p-NRF2, as demonstrated with representative immunofluorescence images (scale bar: 20 μM) and quantified using CellReporterXpress version 2.9 (*p* = 0.028). In addition, metformin increased the gene expression of two other key antioxidant enzymes regulated by NRF2: Superoxide Dismutase 2 (*SOD2*) and Catalase (*CAT*) (*p* = 0.008 and *p* = 0.003, respectively). The protein level of KEAP1 was normalized to β-Actin. Full unedited western blots can be found in Figure S6. (**C**) Overview of the effect of metformin on the antioxidant mechanism. sMaf = small musculoaponeurotic fibrosarcoma, ARE = antioxidant response elements Created in BioRender [52]. The mRNA levels of target genes were normalized to the housekeeping gene TATA Box Binding Protein (*TBP*). The paired *t*-test was used when data were normally distributed (average intensity NQO1, relative protein expression of KEAP1 and % positive nuclei p-NRF2), and the Wilcoxon Signed-Ranks Test was performed when data were not normally distributed (all other markers) to assess differences between the untreated and metformin-treated SMCs. The Mann–Whitney U test was used to compare the NQO1 plasma levels between ND-AAA and D-AAA patients. The data represent mean with standard deviation in the graphs of average intensity NQO1, relative protein expression of KEAP1 and % positive nuclei p-NRF2. Data represent the median and interquartile range in all other graphs. Light blue: expression in AAA samples; dark blue: expression in AAA samples +MET. * *p* ≤ 0.050; ** *p* ≤ 0.010. MET = metformin; a.u. = arbitrary units.

Although metformin did not significantly affect *G6PD* gene expression (*p* = 0.865), it increased the gene expression of the NAD(P)H-generating enzyme *PGD* (*p* = 0.005), as well as the gene expression of the NAD(P)H-dependent antioxidant *CBR1* (*p* = 0.009) (Figure 8A). In addition, protein expression of the NAD(P)H-dependent antioxidant NQO1 was significantly increased after four days of metformin treatment (*p* = 0.001) (Figure 8A). With the exception of G6PD, these findings are consistent with the proteomics analysis, which showed higher expression levels in the SMCs of D-AAA patients using metformin compared to ND-AAA patients. In a genome-wide association meta-analysis, increased plasma levels of the protein NQO1 were associated with a decreased risk of AAA [50], suggesting it may be a potential new therapeutic target for AAA. To test whether metformin affects circulating levels of NQO1, we compared NQO1 enzymatic activity in the plasma of ND-AAA and D-AAA patients. There was a trend toward higher plasma levels in D-AAA patients (*p* = 0.064).

To further assess the protective effect of metformin against oxidative stress, we examined its impact on the expression of key antioxidant regulators (Figure 8B). Six-hour treatment with 10 mM metformin decreased the protein expression of Kelch-Like ECH-Associated Protein 1 (KEAP1) (*p* = 0.047), a repressor of NRF2. Downregulation of KEAP1 is known to reduce the degradation of NRF2, allowing phosphorylated NRF2 (p-NRF2) to translocate to the nucleus, where it forms heterodimers with one of the small musculoaponeurotic fibrosarcoma (sMaf) proteins and binds to regulatory DNA regions called antioxidant response elements (AREs), thereby activating the transcription of genes encoding antioxidant enzymes [51]. Here, we observed increased nuclear p-NRF2 at Ser40 after three days of treatment with 10 mM metformin (*p* = 0.028). Consequently, metformin enhanced the gene expression of two NRF2-downstream key antioxidant regulators: Superoxide Dismutase 2 (*SOD2*) and Catalase (*CAT*) (*p* = 0.008 and *p* = 0.003, respectively). SOD2 facilitates the conversion of Superoxide anion (O_2_^−^) into oxygen (O_2_) and Hydrogen Peroxide (H_2_O_2_), while CAT converts H_2_O_2_ into water (H_2_O) and O_2_ [10]. An overview of the effect of metformin on the antioxidant mechanism is shown in Figure 8C.

## 4. Discussion

To the best of our knowledge, this is the first study to identify significant differences in the proteomic profile of cultured SMCs derived from controls, ND-AAA patients and D-AAA patients. The findings not only highlight distinct biological pathways and molecular functions but also reveal therapeutic targets of metformin that may underlie the inhibition of AAA progression that is observed in D-AAA patients.

Comparison of the proteome of SMCs from ND-AAA patients with controls revealed a reduced expression of proteins involved in metabolic processes and mitochondrial function, with ALDH2 and GYS1 showing consistent changes in aortic tissue. These results align with the findings of a previous study that identified mitochondrial dysfunction in AAA tissue [34,53,54]. Proteins associated with the cytoskeleton and ECM were elevated in ND-AAA-SMCs compared to C-SMCs. The enhanced ECM remodeling response could result from compensation for the increased ECM degradation in the aneurysmal aortic wall. In the comparison between ND-AAA-SMCs and D-AAA-SMCs, a similar cluster of mechanosensitive proteins was observed. Several of these significantly altered proteins are known to be involved in aneurysm formation, as previously reported [30,31,38,39,41,42]. These findings suggest that SMCs from the different patient subgroups retain distinct molecular signatures in vitro, which reliably reflect important features of AAA pathology.

A key finding is that D-AAA-SMCs exhibited an improved metabolic and antioxidant protein profile compared to ND-AAA-SMCs, which may help explain the reported association between diabetes, and in particular metformin use, and reduced AAA growth. Furthermore, the majority of proteins with increased expression in D-AAA-SMCs compared to ND-AAA-SMCs that form a PPI network were confirmed in aortic tissue. Among the KEGG pathways enriched in D-AAA-SMCs relative to ND-AAA-SMCs, the ‘pentose phosphate pathway’, ‘glutathione metabolism’ and ‘chemical carcinogenesis—reactive oxygen species’ reflected increased expression of proteins involved in NAD(P)H generation and its use as a cofactor, both of which contribute to antioxidant defense. Furthermore, the Reactome pathways ‘KEAP1-NFE2L2 pathway’ and ‘nuclear events mediated by NFE2L2’ were enriched. Notably, previous studies have reported that under hyperglycemic conditions, impairment of the KEAP1-NRF2 pathway leads to a decreased expression of antioxidant enzymes, resulting in increased oxidative stress and consequent cellular and organ damage [55]. These findings indicate that metformin use may counteract this pathway and underlie the observed proteomic differences between D-AAA and ND-AAA patients. Since metformin has been shown to alter DNA-methylation in white blood cells [56], it is of interest to study which altered methylation marks in SMCs may explain our distinct signatures.

In vitro studies were performed to investigate whether long-term metformin treatment in patients could explain the proteomic differences observed between ND-AAA and D-AAA SMCs. Typical for diabetes, AGEs are known to be formed. We observed an upregulation of the AGE pentosidine in the cell supernatant after metformin treatment. This aligns with observations by Koole et al. [57], who found that the concentration of pentosidine was higher in aortic biopsies from diabetic patients, regardless of AAA presence. In diabetic AAA patients, elevated pentosidine levels were negatively correlated with aortic diameter [57]. AGEs may help preserve vessel wall structure in AAA, due to collagen crosslinking, making it less prone to degradation [48]. These increased pentosidine levels could indirectly contribute to the reduced *COL1A1* expression upon metformin and reduced ECM-related proteins in D-AAA-SMCs. Enhanced collagen expression represents a wound-healing response, which apparently is diminished upon metformin use. Moreover, metformin increased the levels of *TIMP3*, further stabilizing the ECM.

Our current study also demonstrates that metformin induces a metabolic shift in SMCs from oxidative phosphorylation toward glycolysis, as evidenced by decreased oxygen consumption, increased extracellular acidification and elevated levels of excreted L-lactate. Metformin is proposed to exert a mild and reversible inhibitory effect primarily on mitochondrial respiratory chain complex I, and possibly also on complex IV, thereby reducing oxidative phosphorylation. As a result, cells become more dependent on glycolysis, which may enhance glucose uptake [58]. Inhibition of complex I reduces ATP production and increases the AMP/ATP ratio, leading to AMPK activation, as reflected in our study by an elevated pAMPKα/AMPKα ratio.

AMPK is a well-established activator of PGC-1α, a key regulator of mitochondrial biogenesis [59]. In line with this, we observed increased gene expression of *PGC-1α* and mitochondrial markers *SDHB* and *MT-ATP6*. Notably, we have previously shown that metformin elevates both mitogenic *PPARγ* gene expression and PGC-1α protein expression [8]. In the current study, metformin treatment also led to increased expression of *PPARA* and *HADHB*, two key genes involved in the fatty acid oxidation. Taken together, these findings suggest that metformin stimulates mitochondrial biogenesis, which may be beneficial in AAA, characterized by impaired mitochondrial biogenesis [10,60].

Furthermore, inhibition of complex I leads to an accumulation of NADH and an increase in mitochondrial redox potential (NADH/NAD^+^ ratio). Concurrently, suppression of complex IV may impair mitochondrial glycerol-3-phosphate dehydrogenase (mGPDH), resulting in an elevated cytosolic redox potential [58]. This shift in redox balance may promote NAD(P)H-generating pathways, such as the pentose phosphate pathway, where PGD contributes to NAD(P)H production. It may also explain the increased expression of *NQO1* and *CBR1*, both of which use NAD(P)H as a cofactor in antioxidant defense. Consistent with this, our previous study [8] demonstrated enhanced activity of NAD(P)/NAD(P)H-dependent oxidoreductase enzymes. These observations highlight the effect of metformin on redox balance, which may underlie the triggered compensatory mechanisms beneficial for SMC health. In this light, it is of interest that boosting NAD levels with its precursor nicotinamide riboside reduced AAA formation and prevented aortic rupture in a murine AAA model [54].

Both AMPK and NRF2 are activated in response to cellular stress, and although their interaction is not yet fully understood, it is assumed that these pathways may be interdependent and cooperate to restore cellular homeostasis [61]. In this study, following metformin treatment, we observed reduced KEAP1 levels and consequently enhanced nuclear localization of p-NRF2, which activates the antioxidant defense response. This activation is reflected by elevated expression levels of NRF2 targets genes, including SOD2, CAT, CBR1, PGD, and NQO1, in AAA-SMCs upon metformin treatment. NRF2 deficiency has been reported to contribute to AAA formation [62,63], whereas enhanced expression of SOD2 or CAT has shown protective effects against AAA development in animal models [64,65]. Moreover, activation of the NRF2 pathway and subsequent increased expression of NQO1 inhibited the conversion of contractile SMCs to the synthetic phenotype [66,67]. Notably, NQO1 was identified as the hub protein in the cluster of proteins with increased expression in D-AAA-SMCs compared to ND-AAA-SMCs. Considering the reported inverse association of NQO1 expression with AAA growth [50], NQO1 may hold potential as a risk biomarker, which should be evaluated prospectively.

Several attempts have been made to enhance antioxidant defense using exogenous compounds, such as α-tocopherol (a form of vitamin E) and β-carotene (vitamin A), to inhibit AAA progression [10]. However, although many promising results from preclinical studies failed to translate into clinical success, clinical data indicate a reduced prevalence and slower growth rate of AAA in diabetic patients, particularly those using metformin [5]. The strength of the effect of metformin is likely multifactorial, impacting various processes through the regulation of specific metabolic pathways that influence redox balance, energy metabolism and inflammatory responses [1,8]. Randomized controlled trials have been initiated to evaluate metformin’s protective effect on aneurysm progression in non-diabetic AAA patients, with the goal of providing level I evidence [68].

To identify potential therapeutic targets to inhibit AAA progression, it may be more effective to study AAA subgroups where the outcome is known to impact AAA progression, rather than comparing non-diseased aortic tissue with end-stage AAA tissue.

Several limitations should be acknowledged. Hypertension and renal dysfunction were more prevalent in D-AAA patients than in ND-AAA patients, which may have affected the SMC proteomic findings. However, in the tissue proteomics dataset used to validate several key findings, these baseline characteristics were comparable between the two groups, indicating that the observed differences in expression of validated proteins are unlikely to be driven by these comorbidities.

Furthermore, the number of diabetic AAA patients included in the SMC proteomics analysis was relatively small. Despite this limitation, their proteomic profile revealed a unique signature that distinguished them from the other study groups. To enhance robustness, we applied a 75% data presence filter, thereby concentrating on consistently discriminating proteins. Rather than examining single proteins, we focused on clusters of proteins participating in the same biological pathways and processes. Moreover, we validated the key findings in a tissue proteomics dataset with a larger sample size, supporting the reproducibility of the results. Importantly, oxidoreductase-related proteins showed similar expression patterns in both SMCs in vitro and aortic tissue samples, reinforcing the biological significance of our findings and confirming that these changes are not generated by the in vitro environment.

Another limitation is that we used a relatively high metformin concentration (10 mM) in cell culture experiments, which is far above physiological plasma levels observed in patients. This was chosen to induce rapid effects on SMCs without causing cell death. While this approach allowed the detection of cellular responses within a short timeframe, in vitro data are not directly translatable to the clinical setting, due to differences in physiological conditions and the long-term, lower-dose use of metformin in vivo. Moreover, the possibility of off-target effects at this concentration cannot be ruled out. However, for many drugs, short-term high-dose effects in cell cultures correspond to prolonged low-dose treatment in vivo [8].

Furthermore, we only investigated differences in proteomic profile in SMCs of ND-AAA patients and D-AAA patients all using metformin in the SMC proteomics analysis. We did not include D-AAA patients receiving other diabetes treatments, as we did not have sufficient patient SMCs from this subgroup. Nevertheless, metformin is the only drug that has shown a significant inhibition of AAA progression [3]. We acknowledge that using SMCs from D-AAA patients all treated with metformin limits our ability to distinguish diabetes-specific effects from potential metformin-induced effects. To clarify whether the observed effects are specifically due to metformin, it would be valuable to include SMCs from diabetic patients not receiving any diabetes treatment. However, we attempted to address this limitation by specifically investigating the effect of metformin in vitro on SMCs derived from AAA patients, which provided additional insight into its impact on the identified pathways and processes.

## 5. Conclusions

This study identified significant differences in the proteome of SMCs derived from controls, ND-AAA and D-AAA patients. It highlights distinct pathways in relation to mechanosensing, metabolism and redox balance as therapeutic targets of metformin that may underlie its inhibition of AAA progression.

## Figures and Tables

**Table 1:** **Clinical characteristics of aortic controls (*n* = 8), non-diabetic (*n* = 19) and diabetic (*n* = 5) abdominal aortic aneurysm patients.**

	Control (*n* = 8)	ND-AAA (*n* = 19)	D-AAA (*n* = 5) *	*p* Value C vs. ND-AAA vs. D-AAA	*p* Value ND-AAA vs. D-AAA
Age (years)	52.4 ± 16.7	72.1 ± 9.1	75.0 ± 7.8	<0.001	0.520
Male	3 (42.9)	12 (66.7)	2 (40)	0.893	1.000
Aneurysm size (mm)	N/A	76.2 ± 19.9	65.0 ± 10.1	N/A	0.113
Rupture	N/A	3 (16.7)	1 (20.0)	N/A	1.000
Current smoking	N/A	6 (35.3)	0 (0.0)	N/A	0.266
Hypertension †	N/A	7 (43.8)	5 (100)	N/A	0.045
Previous vascular surgery ‡	N/A	6 (37.5)	3 (60)	N/A	0.611
Renal dysfunction	N/A	1 (6.3)	4 (80)	N/A	0.004
BMI	N/A	26.2 ± 4.1	29.2 ± 6.3	N/A	0.211

Data are presented as *n* (%) or mean ± standard deviation. Valid percentages were reported in cases of missing clinical data. AAA = abdominal aortic aneurysm; ND = non-diabetic; D = diabetic; BMI = body mass index; N/A = not applicable; * all diabetic AAA patients used metformin; † hypertension and renal dysfunction were defined as diagnosed by a medical doctor or using specific medication. The renal function of controls is presumed to be sufficient, as they have been approved for kidney donation; ‡ previous vascular surgery includes percutaneous coronary intervention, percutaneous transluminal angioplasty, coronary artery bypass grafting and endovascular aneurysm repair.

## Data Availability

The original mass spectrometry proteomics data presented in this study are openly available, as they have been deposited to the ProteomeXchange consortium via the PRIDE (Proteomics Identifications) [22] partner repository with the SMC dataset identifier PXD054353 and the tissue dataset identifier PXD067859.

## Supplementary Materials

The following supporting information can be downloaded at: https://www.mdpi.com/article/10.3390/medsci13030184/s1, **Figure S1.** Coomassie blue staining of samples for quality control; **Figure S2.** Protein-protein interaction (PPI) network analyses performed for the proteins with increased and decreased expression in ND-AAA-SMC compared to C-SMC. Proteins with lower expression in ND-AAA-SMCs highlighted in yellow, and proteins with higher expression in ND-AAA-SMCs compared to C-SMCs highlighted in green. Proteins that are part of the cluster, since the proteins with increased and decreased expression were taken together, are highlighted with a thick outline. **Figure S3. Protein-Protein Interaction (PPI) network analyses performed for the proteins with increased and decreased expression in D-AAA-SMC compared to ND-AAA-SMC**. Proteins with lower expression in D-AAA-SMCs highlighted in green, and proteins with higher expression in D-AAA-SMCs compared to ND-AAA-SMCs highlighted in blue. Proteins that have now become part of the integrated cluster of the proteins with increased and decreased expression together, are highlighted with a thick outline. **Figure S4. Expression of hub proteins in aortic SMC derived from diabetic AAA patients compared to non-diabetic AAA patients. A.** Protein expression of the proteins that were decreased in D-AAA-SMCs compared to ND-AAA-SMCs. Filamin A (FLNA) (log2 (Fold Change(FC)) = −1.17, *p* = 0.025), Fibronectin 1 (FN1) (log2(FC) = −1.46, *p* = 0.036), and Actinin Alpha 1 (ACTN1) (log2(FC) = −1.20, *p* = 0.032). In addition, there was a significant difference comparing the expression of ACTN1 between SMCs of controls (C) and ND-AAA patients (log2(FC) = 1.13, *p* = 0.028). **B.** Protein expression of the proteins that were increased in D-AAA-SMCs compared to ND-AAA-SMCs. NAD(P)H: Quinone Oxidoreductase 1 (NQO1) (log2(FC) = 2.94, *p* = 0.019), Glucose-6-Phosphate Dehydrogenase (G6PD) (log2(FC) = 1.24, *p* = 0.026), and 6-Phosphogluconate Dehydrogenase (PGD) (log2(FC) = 1.52, *p* = 0.047). The counts of the proteomics data shown in A and B were normalized to the total spectral count per sample. The proteomics spectral count data were tested using a β-binomial test for independent samples. Data represent the median and interquartile range, ∗ *p* ≤ 0.050; ∗∗ *p* ≤ 0.010. **Figure S5. The effect of metformin on the cytoskeleton and extracellular matrix in aortic SMC derived from AAA patients. A.** Metformin treatment had no significant effect on the gene expression of Filamin A (*FLNA*) and Actinin Alpha 1 (*ACTN1*) (*p* = 0.972 and *p* = 0.245, respectively). However, metformin decreased the gene expression of cytoskeleton marker Myosin Light Chain 12A (*MYL12A*), in SMCs of AAA patients (*p* = 0.015). **B.** Metformin treatment had no significant effect on the gene expression of Fibronectin 1 (*FN1*) (*p* = 0.975). Moreover, metformin decreased the gene expression of two other extracellular matrix genes, Tissue Inhibitor of Metalloproteinases 3 (*TIMP3*) and Collagen Type I alpha 1 (*COL1A1*) (*p* = 0.005 and *p* = 0.013, respectively). The formation of Pentosidine, an Advanced Glycation End Product, was increased in cell supernatants after metformin treatment (*p* = 0.050). The mRNA levels of target genes were normalized to the housekeeping gene TATA box Binding Protein (*TBP*). The Wilcoxon Signed-Ranks Test was performed to assess differences between the untreated and metformin treated SMCs. Data represent the median and interquartile range. ∗ *p* ≤ 0.050; ∗∗ *p* ≤ 0.010. MET = metformin. **Figure S6. Full unedited western blots. The expression was measured in six different SMC lines derived from AAA patients, untreated (u) and after metformin treatment (M).** **Table S1**: Primer sequences of genes that were assayed by Quantitative Polymerase Chain Reaction. **Table S2. Clinical characteristics of aortic controls (*n* = 17), non-diabetic (*n* = 42) and diabetic (*n* = 15) abdominal aortic aneurysm patients.** Data are presented as *n* (%) or mean ± standard deviation. Valid percentages were reported in cases of missing clinical data. AAA = abdominal aortic aneurysm; ND = non-diabetic; D = diabetic; BMI = Body Mass Index; N/A = not applicable. * Eight diabetic AAA patients used metformin. Of the seven non-metformin D-AAA patients not using metformin, three were not on antidiabetic medication, two used dapagliflozin, one used tolbutamide, and one used insulin. †Hypertension and renal dysfunction were defined as diagnosed by a medical doctor or using specific medication. The renal function of controls is presumed to be sufficient, as they have been approved for kidney donation. ‡ Previous vascular surgery includes percutaneous coronary intervention, percutaneous transluminal angioplasty, coronary artery bypass grafting and endovascular aneurysm repair. **Table S3. SMC protein report. Table S4. Significantly differentially expressed proteins after in aortic SMC derived from non-diabetic AAA patients (ND-AAA) (*n* = 19), diabetic AAA patients (D-AAA) (*n* = 5) and aortic controls (C) (*n* = 8).** An log2(fold change (FC)) of ∞ signifies that the protein was found in one or two of the three study groups, but was absent in the others. **Table S5. Significantly differentially expressed proteins after applying a 50% data presence filter in aortic SMC derived from non-diabetic AAA patients (ND-AAA) (*n* = 19) compared to aortic controls (C) (*n* = 8).** A negative log2(fold change (FC)) indicates the protein expression was lower (L) in ND-AAA-SMC compared to C-SMC, while a positive log2(FC) indicates that the protein expression was higher (H) in ND-AAA-SMC compared to C-SMC. Additionally, an log2(FC) of −∞ or ∞ signifies that the protein was exclusively detected in the C-SMC or ND-AAA-SMC, respectively. **Table S6. Identifiers of the KEGG, Reactome and GO terms.** **Table S7. Significantly differentially expressed proteins after applying a 75% data presence filter in aortic SMC derived from diabetic AAA patients (D-AAA) (*n* = 5) compared to non-diabetic AAA patients (ND-AAA) (*n* = 19).** A negative log2(fold change (FC)) indicates the protein expression was lower (L) in D-AAA-SMC compared to ND-AAA-SMC, while a positive log2(FC) indicates that the protein expression was higher (H) in D-AAA-SMC compared to ND-AAA-SMC. Additionally, an log2(FC) of −∞ or ∞ signifies that the protein was exclusively detected in the ND-AAA-SMC or D-AAA-SMC, respectively. **Table S8. Tissue protein report.**

## Abbreviations/Nomenclature

AAA	Abdominal aortic aneurysm
ACTG1	Actin Gamma-1
ACTN1	Actinin Alpha 1
ADAM10	A Disintegrin and Metalloproteinase 10
AGEs	Advanced Glycation End Products
AKR	Aldo-Keto Reductases
ALDH2	Aldehyde Dehydrogenase 2 Family Member
AMPK	AMP-Activated Protein Kinase
ARE	Antioxidant response elements
C	Non-pathological controls (postmortem heart-beating kidney donors)
CAT	Catalase
CBR1	Carbonyl Reductase 1
CNN2	Calponin 2
COL1A1	Collagen Type I Alpha 1
COL5A1	Collagen Type V Alpha-1
COLGALT1	Collagen Beta(1-O)Galactosyltransferase 1
CTHRC1	Collagen Triple Helix Repeat Containing 1
D-AAA	diabetic AAA patients
ECAR	Extracellular acidification rate
ECM	Extracellular matrix
EML4	Echinoderm Microtubule-Associated Protein-Like 4
EPHX1	Epoxide Hydrolase 1
EVAR	Endovascular aneurysm repair
EZR	Ezrin
FDR	False Discovery Rate
FLNA	Filamin A
FLNB	Filamin B
FLNC	Filamin C
FN1	Fibronectin 1
G6PD	Glucose-6-Phosphate Dehydrogenase
GO	Gene Ontology
GSTM1	Glutathione-S-Transferase M1
GYS1	Glycogen Synthase 1
H_2_O_2_	Hydrogen Peroxide
HADHB	Hydroxyacyl-CoA Dehydrogenase Trifunctional Multienzyme Complex Subunit Beta
KEAP1	Kelch-Like ECH-Associated Protein 1
KEGG	Kyoto Encyclopedia of Genes and Genomes
LAMC1	Laminin Subunit Gamma-1
mGPDH	Mitochondrial glycerol-3-phosphate dehydrogenase
MMP	Matrix metalloproteinase
MT-ATP6	Mitochondrially Encoded ATP Synthase Membrane Subunit 6
MTP	Mitochondrial Trifunctional Protein
MYL12A	Myosin Light Chain 12A
ND-AAA	Non-diabetic AAA patients
NQO1	NAD(P)H: Quinone Oxidoreductase 1
NRF2	Nuclear Factor Erythroid 2-Related Factor 2
O_2_^−^	Superoxide anion
OCR	Oxygen consumption rate
PCK2	Phosphoenolpyruvate Carboxykinase 2
PDLIM1	PDZ and LIM Domain Protein 1
PGC-1α	Peroxisome Proliferator-Activated Receptor Gamma Coactivator 1-Alpha
PGD	6-Phosphogluconate Dehydrogenase
PGM2	Phosphoglucomutase 2
PPARA	Peroxisome Proliferator-Activated Receptor Alpha
PPI	Protein–protein interaction
PRKG1	Protein Kinase cGMP-Dependent 1
RNS	Reactive nitrogen species
ROS	Reactive oxygen species
RT-qPCR	Real-time quantitative Polymerase Chain Reaction
SDHB	Succinate Dehydrogenase Complex Subunit B
sMaF	Small Maf proteins
SMC	Smooth muscle cell
SOD2	Superoxide Dismutase 2
T2D	Type 2 diabetes
TAGLN2	Transgelin-2
TBP	TATA box binding protein
TFA	Trifluoroacetic acid
TGFBI	Growth Factor Beta-Induced Protein
TIMP3	Tissue Inhibitor of Metalloproteinase 3
TKT	Transketolase

## References

[1] Yang L., Shen L., Gao P., Li G., He Y., Wang M., Zhou H., Yuan H., Jin X., Wu X. (2017). Effect of AMPK signal pathway on pathogenesis of abdominal aortic aneurysms. Oncotarget.

[2] Jessula S., Cote C.L., Cooper M., McDougall G., Kivell M., Kim Y., Tansley G., Casey P., Smith M., Herman C. (2023). Dying to Get There: Patients Who Reside at Increased Distance from Tertiary Center Experience Increased Mortality Following Abdominal Aortic Aneurysm Rupture. Ann. Vasc. Surg..

[3] Golledge J., Moxon J., Pinchbeck J., Anderson G., Rowbotham S., Jenkins J., Bourke M., Bourke B., Dear A., Buckenham T. (2017). Association between metformin prescription and growth rates of abdominal aortic aneurysms. Brit J. Surg..

[4] Wanhainen A., Van Herzeele I., Bastos Goncalves F., Bellmunt Montoya S., Berard X., Boyle J.R., D’Oria M., Prendes C.F., Karkos C.D., Kazimierczak A. (2024). Editor’s Choice—European Society for Vascular Surgery (ESVS) 2024 Clinical Practice Guidelines on the Management of Abdominal Aorto-Iliac Artery Aneurysms. Eur. J. Vasc. Endovasc. Surg..

[5] Kristensen J.S.S., Krasniqi L., Obel L.M., Kavaliunaite E., Liisberg M., Lindholt J.S. (2024). Exploring Drug Re-Purposing for Treatment of Abdominal Aortic Aneurysms: A Systematic Review and Meta-analysis. Eur. J. Vasc. Endovasc. Surg..

[6] Itoga N.K., Rothenberg K.A., Suarez P., Ho T.V., Mell M.W., Xu B., Curtin C.M., Dalman R.L. (2019). Metformin prescription status and abdominal aortic aneurysm disease progression in the U.S. veteran population. J. Vasc. Surg..

[7] van Merrienboer T.A.R., Warlich V., Holewijn S., Driessen W., Yeung K.K., Reijnen M. (2025). The Impact of Diabetes Mellitus and Metformin Use on Outcomes After Endovascular Aneurysm Repair. J. Clin. Med..

[8] van Merrienboer T.A.R., Rombouts K.B., Bogunovic N., Mieremet A., Meekel J.P., Balm R., de Waard V., Yeung K.K. (2024). Metformin Improves the Function of Abdominal Aortic Aneurysm Patient Derived Aortic Smooth Muscle Cells. Eur. J. Vasc. Endovasc. Surg..

[9] Summerhill V.I., Sukhorukov V.N., Eid A.H., Nedosugova L.V., Sobenin I.A., Orekhov A.N. (2021). Pathophysiological Aspects of the Development of Abdominal Aortic Aneurysm with a Special Focus on Mitochondrial Dysfunction and Genetic Associations. Biomol. Concepts.

[10] Kazaleh M., Gioscia-Ryan R., Ailawadi G., Salmon M. (2023). Oxidative Stress and the Pathogenesis of Aortic Aneurysms. Biomedicines.

[11] Xiong W., Mactaggart J., Knispel R., Worth J., Zhu Z., Li Y., Sun Y., Baxter B.T., Johanning J. (2009). Inhibition of reactive oxygen species attenuates aneurysm formation in a murine model. Atherosclerosis.

[12] Oller J., Gabande-Rodriguez E., Ruiz-Rodriguez M.J., Desdin-Mico G., Aranda J.F., Rodrigues-Diez R., Ballesteros-Martinez C., Blanco E.M., Roldan-Montero R., Acuna P. (2021). Extracellular Tuning of Mitochondrial Respiration Leads to Aortic Aneurysm. Circulation.

[13] Rombouts K.B., van Merrienboer T.A.R., Henneman A.A., Knol J.C., Pham T.V., Piersma S.R., Jimenez C.R., Bogunovic N., van der Velden J., Yeung K.K. (2024). Insight in the (Phospho)proteome of Vascular Smooth Muscle Cells Derived From Patients With Abdominal Aortic Aneurysm Reveals Novel Disease Mechanisms. Arterioscler. Thromb. Vasc. Biol..

[14] Pham T.V., Piersma S.R., Warmoes M., Jimenez C.R. (2010). On the beta-binomial model for analysis of spectral count data in label-free tandem mass spectrometry-based proteomics. Bioinformatics.

[15] Bardou P., Mariette J., Escudié F., Djemiel C., Klopp C. (2014). jvenn: An interactive Venn diagram viewer. BMC Bioinform..

[16] Ge S.X., Jung D.M., Yao R.A. (2020). ShinyGO: A graphical gene-set enrichment tool for animals and plants. Bioinformatics.

[17] Kanehisa M., Furumichi M., Sato Y., Ishiguro-Watanabe M., Tanabe M. (2021). KEGG: Integrating viruses and cellular organisms. Nucleic Acids Res..

[18] Tang D.D., Chen M.J., Huang X.H., Zhang G.C., Zeng L., Zhang G.S., Wu S.J., Wang Y.W. (2023). SRplot: A free online platform for data visualization and graphing. PLoS ONE.

[19] Szklarczyk D., Kirsch R., Koutrouli M., Nastou K., Mehryary F., Hachilif R., Gable A.L., Fang T., Doncheva N.T., Pyysalo S. (2023). The STRING database in 2023: Protein-protein association networks and functional enrichment analyses for any sequenced genome of interest. Nucleic Acids Res..

[20] Shannon P., Markiel A., Ozier O., Baliga N.S., Wang J.T., Ramage D., Amin N., Schwikowski B., Ideker T. (2003). Cytoscape: A software environment for integrated models of biomolecular interaction networks. Genome Res..

[21] Chin C.H., Chen S.H., Wu H.H., Ho C.W., Ko M.T., Lin C.Y. (2014). *CytoHubba*: Identifying hub objects and sub-networks from complex interactome. BMC Syst. Biol..

[22] Perez-Riverol Y., Bai J., Bandla C., Garcia-Seisdedos D., Hewapathirana S., Kamatchinathan S., Kundu D.J., Prakash A., Frericks-Zipper A., Eisenacher M. (2022). The PRIDE database resources in 2022: A hub for mass spectrometry-based proteomics evidences. Nucleic Acids Res..

[23] Zougman A., Selby P.J., Banks R.E. (2014). Suspension trapping (STrap) sample preparation method for bottom-up proteomics analysis. Proteomics.

[24] Thanou E., Koopmans F., Pita-Illobre D., Klaassen R.V., Ozer B., Charalampopoulos I., Smit A.B., Li K.W. (2023). Suspension TRAPping Filter (sTRAP) Sample Preparation for Quantitative Proteomics in the Low microg Input Range Using a Plasmid DNA Micro-Spin Column: Analysis of the Hippocampus from the 5xFAD Alzheimer’s Disease Mouse Model. Cells.

[25] Demichev V., Messner C.B., Vernardis S.I., Lilley K.S., Ralser M. (2020). DIA-NN: Neural networks and interference correction enable deep proteome coverage in high throughput. Nat. Methods.

[26] Pham T.V., Henneman A.A., Jimenez C.R. (2020). iq: An R package to estimate relative protein abundances from ion quantification in DIA-MS-based proteomics. Bioinformatics.

[27] Tsai S.H., Hsu L.A., Tsai H.Y., Yeh Y.H., Lu C.Y., Chen P.C., Wang J.C., Chiu Y.L., Lin C.Y., Hsu Y.J. (2020). Aldehyde dehydrogenase 2 protects against abdominal aortic aneurysm formation by reducing reactive oxygen species, vascular inflammation, and apoptosis of vascular smooth muscle cells. FASEB J..

[28] Gautheron J., Jéru I. (2021). The Multifaceted Role of Epoxide Hydrolases in Human Health and Disease. Int. J. Mol. Sci..

[29] Annabi B., Shedid D., Ghosn P., Kenigsberg R.L., Desrosiers R.R., Bojanowski M.W., Beaulieu E., Nassif E., Moumdjian R., Beliveau R. (2002). Differential regulation of matrix metalloproteinase activities in abdominal aortic aneurysms. J. Vasc. Surg..

[30] Carrell T.W.G., Burnand K.G., Wells G.M.A., Clements J.M., Smith A. (2002). Stromelysin-1 (matrix metalloproteinase-3) and tissue inhibitor of metalloproteinase-3 are overexpressed in the wall of abdominal aortic aneurysms. Circulation.

[31] Basu R., Fan D., Kandalam V., Lee J., Das S.K., Wang X., Baldwin T.A., Oudit G.Y., Kassiri Z. (2012). Loss of Timp3 gene leads to abdominal aortic aneurysm formation in response to angiotensin II. J. Biol. Chem..

[32] Cao M., Ke D., Zhou H. (2024). The role and molecular mechanism of CTHRC1 in fibrosis. Life Sci..

[33] Modrego J., Lopez-Farre A.J., Martinez-Lopez I., Muela M., Macaya C., Serrano J., Monux G. (2012). Expression of cytoskeleton and energetic metabolism-related proteins at human abdominal aortic aneurysm sites. J. Vasc. Surg..

[34] Gabel G., Northoff B.H., Balboa A., Becirovic-Agic M., Petri M., Busch A., Maegdefessel L., Mahlmann A., Ludwig S., Teupser D. (2021). Parallel Murine and Human Aortic Wall Genomics Reveals Metabolic Reprogramming as Key Driver of Abdominal Aortic Aneurysm Progression. J. Am. Heart Assoc..

[35] Milewicz D.M., Guo D., Hostetler E., Marin I., Pinard A.C., Cecchi A.C. (2021). Update on the genetic risk for thoracic aortic aneurysms and acute aortic dissections: Implications for clinical care. J. Cardiovasc. Surg..

[36] Milewicz D.M., Guo D.C., Tran-Fadulu V., Lafont A.L., Papke C.L., Inamoto S., Kwartler C.S., Pannu H. (2008). Genetic basis of thoracic aortic aneurysms and dissections: Focus on smooth muscle cell contractile dysfunction. Annu. Rev. Genomics Hum. Genet..

[37] Zheng Y., Ma H., Yan Y., Ye P., Yu W., Lin S., Chen S.L. (2023). Deficiency of filamin A in smooth muscle cells protects against hypoxia-mediated pulmonary hypertension in mice. Int. J. Mol. Med..

[38] Chen M.H., Deng E.S., Yamada J.M., Choudhury S., Scotellaro J., Kelley L., Isselbacher E., Lindsay M.E., Walsh C.A., Doan R.N. (2024). Contributions of Germline and Somatic Mosaic Genetics to Thoracic Aortic Aneurysms in Nonsyndromic Individuals. J. Am. Heart Assoc..

[39] Pinard A., Jones G.T., Milewicz D.M. (2019). Genetics of Thoracic and Abdominal Aortic Diseases Aneurysms, Dissections, and Ruptures. Circ. Res..

[40] Jain M., Chauhan A.K. (2022). Role of Integrins in Modulating Smooth Muscle Cell Plasticity and Vascular Remodeling: From Expression to Therapeutic Implications. Cells.

[41] Qiu R.F., Chen S.X., Gao P.X., Luo K., Feng X.D., Yuan H., Wu X.J., Li G. (2021). ADAM10 attenuates the development of abdominal aortic aneurysms in a mouse model. Mol. Med. Rep..

[42] Chen P., Yu B., Li Z.Z., Chen Y.H., Sun Y., Wang D.W. (2021). Variants Cause Aortic Dissection by Activating TGF-β-Signaling Pathway. J. Am. Heart Assoc..

[43] Hamann B., Klimova A., Klotz F., Frank F., Janichen C., Kapalla M., Sabarstinski P., Wolk S., Morawietz H., Poitz D.M. (2023). Regulation of CD163 Receptor in Patients with Abdominal Aortic Aneurysm and Associations with Antioxidant Enzymes HO-1 and NQO1. Antioxidants.

[44] Hayes J.D., Dinkova-Kostova A.T. (2014). The Nrf2 regulatory network provides an interface between redox and intermediary metabolism. Trends Biochem. Sci..

[45] Song N., Yu J.E., Ji E., Choi K.H., Lee S. (2024). Hydrogen sulfide inhibits gene expression associated with aortic valve degeneration by inducing NRF2-related pro-autophagy effect in human aortic valve interstitial cells. Mol. Cell Biochem..

[46] Kruger A., Gruning N.M., Wamelink M.M., Kerick M., Kirpy A., Parkhomchuk D., Bluemlein K., Schweiger M.R., Soldatov A., Lehrach H. (2011). The pentose phosphate pathway is a metabolic redox sensor and regulates transcription during the antioxidant response. Antioxid. Redox Signal.

[47] Heiss E.H., Schachner D., Zimmermann K., Dirsch V.M. (2013). Glucose availability is a decisive factor for Nrf2-mediated gene expression. Redox Biol..

[48] Dattani N., Sayers R.D., Bown M.J. (2018). Diabetes mellitus and abdominal aortic aneurysms: A review of the mechanisms underlying the negative relationship. Diab. Vasc. Dis. Res..

[49] van der Pluijm I., Burger J., van Heijningen P.M., Ijpma A., van Vliet N., Milanese C., Schoonderwoerd K., Sluiter W., Ringuette L.J., Dekkers D.H.W. (2018). Decreased mitochondrial respiration in aneurysmal aortas of Fibulin-4 mutant mice is linked to PGC1A regulation. Cardiovasc. Res..

[50] Roychowdhury T., Klarin D., Levin M.G., Spin J.M., Rhee Y.H., Deng A., Headley C.A., Tsao N.L., Gellatly C., Zuber V. (2023). Genome-wide association meta-analysis identifies risk loci for abdominal aortic aneurysm and highlights PCSK9 as a therapeutic target. Nat. Genet..

[51] Averill-Bates D. (2024). Reactive oxygen species and cell signaling. Review. Biochim. Biophys. Acta Mol. Cell Res..

[52] De Waard V. Created in Biorender. https://BioRender.com/q50d341.

[53] Tavris B.S., Peters A.S., Bockler D., Dihlmann S. (2023). Mitochondrial Dysfunction and Increased DNA Damage in Vascular Smooth Muscle Cells of Abdominal Aortic Aneurysm (AAA-SMC). Oxid. Med. Cell Longev..

[54] Oller J., Gabande-Rodriguez E., Roldan-Montero R., Ruiz-Rodriguez M.J., Redondo J.M., Martin-Ventura J.L., Mittelbrunn M. (2022). Rewiring Vascular Metabolism Prevents Sudden Death due to Aortic Ruptures-Brief Report. Arterioscler. Thromb. Vasc. Biol..

[55] Yi M., Cruz Cisneros L., Cho E.J., Alexander M., Kimelman F.A., Swentek L., Ferrey A., Tantisattamo E., Ichii H. (2024). Nrf2 Pathway and Oxidative Stress as a Common Target for Treatment of Diabetes and Its Comorbidities. Int. J. Mol. Sci..

[56] Li M., Bao L., Zhu P., Wang S. (2022). Effect of metformin on the epigenetic age of peripheral blood in patients with diabetes mellitus. Front. Genet..

[57] Koole D., van Herwaarden J.A., Schalkwijk C.G., Lafeber F., Vink A., Smeets M.B., Pasterkamp G., Moll F.L. (2017). A potential role for glycated cross-links in abdominal aortic aneurysm disease. J. Vasc. Surg..

[58] Foretz M., Guigas B., Viollet B. (2023). Metformin: Update on mechanisms of action and repurposing potential. Nat. Rev. Endocrinol..

[59] Garcia D., Shaw R.J. (2017). AMPK: Mechanisms of Cellular Energy Sensing and Restoration of Metabolic Balance. Mol. Cell.

[60] Gabrielson M., Vorkapic E., Folkesson M., Welander M., Matussek A., Dimberg J., Lanne T., Skogberg J., Wagsater D. (2016). Altered PPARgamma Coactivator-1 Alpha Expression in Abdominal Aortic Aneurysm: Possible Effects on Mitochondrial Biogenesis. J. Vasc. Res..

[61] Petsouki E., Cabrera S.N.S., Heiss E.H. (2022). AMPK and NRF2: Interactive players in the same team for cellular homeostasis?. Free Radic. Biol. Med..

[62] Song H.Y., Xu T., Feng X.F., Lai Y.X., Yang Y., Zheng H., He X., Wei G.Q., Liao W.J., Liao Y.L. (2020). Itaconate prevents abdominal aortic aneurysm formation through inhibiting inflammation via activation of Nrf2. Ebiomedicine.

[63] Kopacz A., Werner E., Grochot-Przeczek A., Kloska D., Hajduk K., Neumayer C., Jozkowicz A., Piechota-Polanczyk A. (2020). Simvastatin Attenuates Abdominal Aortic Aneurysm Formation Favoured by Lack of Nrf2 Transcriptional Activity. Oxid. Med. Cell Longev..

[64] Yu Z.H., Morimoto K., Yu J., Bao W.L., Okita Y., Okada K. (2016). Endogenous superoxide dismutase activation by oral administration of riboflavin reduces abdominal aortic aneurysm formation in rats. J. Vasc. Surg..

[65] Parastatidis I., Weiss D., Joseph G., Taylor W.R. (2013). Overexpression of catalase in vascular smooth muscle cells prevents the formation of abdominal aortic aneurysms. Arterioscler. Thromb. Vasc. Biol..

[66] He X., Deng J., Yu X.J., Yang S., Yang Y., Zang W.J. (2020). Activation of M3AChR (Type 3 Muscarinic Acetylcholine Receptor) and Nrf2 (Nuclear Factor Erythroid 2-Related Factor 2) Signaling by Choline Alleviates Vascular Smooth Muscle Cell Phenotypic Switching and Vascular Remodeling. Arterioscler. Thromb. Vasc. Biol..

[67] Xiao X.Y., Li C.L., Huang X.J., Chen G.A., Huang X.R., Song F.E., Zhou Y., Liu X.C., Zhou X.K., Meng J.X. (2024). Single-cell RNA sequencing reveals that NRF2 regulates vascular smooth muscle cell phenotypic switching in abdominal aortic aneurysm. FASEB J..

[68] Wanhainen A., Dalman R.L. (2025). Update on Ongoing Randomised Controlled Trials Evaluating the Protective Effect of Metformin on Abdominal Aortic Aneurysm Progression. Eur. J. Vasc. Endovasc. Surg..

