# Supplementary material for: Proteome Differences in Smooth Muscle Cells from Diabetic and Non-Diabetic Abdominal Aortic Aneurysm Patients Reveal Metformin-Induced Mechanisms
Source: Med Sci (Basel). 2025 Sep 10;13(3):184. doi: 10.3390/medsci13030184 (PMC12452731; doi:10.3390/medsci13030184)
Supplement: Supplementary file 1 [file medsci-13-00184-s001.zip › medsci-3786036-supplementary.pdf]

Supplemental Material

Proteome Differences in Smooth Muscle Cells from Diabetic and Non-Diabetic Abdominal Aortic Aneurysm Patients Reveal Metformin-Induced Mechanisms

Tara A.R. van Merrienboer [1,2,3], Karlijn B. Rombouts [3,4], Albert C.W.A. van Wijk[1,3,4], Jaco C. Knol[5,6], Thang V. Pham[5,6], Sander R. Piersma[5,6], Connie R. Jimenez[5,6], Ron Balm[1,3], Kak K. Yeung [1,3,4†], Vivian de Waard[2,3†]

1. Amsterdam UMC location University of Amsterdam, Surgery, Meibergdreef 9, Amsterdam, The Netherlands

2. Amsterdam UMC location University of Amsterdam, Medical Biochemistry, Meibergdreef 9, Amsterdam, The Netherlands

3. Amsterdam Cardiovascular Sciences, Atherosclerosis and Aortic Diseases, Amsterdam, The Netherlands

4. Amsterdam UMC location Vrije Universiteit Amsterdam, Physiology, De Boelelaan 1117, Amsterdam, The Netherlands

5. Amsterdam UMC location Vrije Universiteit Amsterdam, Medical Oncology, OncoProteomics Laboratory, Van der Boechorststraat 6, Amsterdam, The Netherlands

6. Amsterdam UMC location Vrije Universiteit Amsterdam, Proteomics Core Resource, Van der Boechorststraat 6, Amsterdam, The Netherlands

[†]Contributed equally as senior authors

Short title: Proteome Differences in SMCs from ND-AAA and D-AAA patients

Supplemental Methods
Major Resources Table
Figure S1-S6
Table S1-S8 (with Table S3 and S8 in separate Excel files)

Supplemental Methods

Patient samples

Patient Population

For this study, blood samples and aortic biopsies were obtained from patients who underwent open repair surgery for AAA between 2016 and 2024 at Amsterdam UMC or Dijklander Hospital, both located in the Netherlands. Before surgery, all patients provided informed consent for their blood and tissue to be stored in the Biobank for Aortic Aneurysms, Atherosclerosis, and Biomarkers (TcB: 2017.121). Control aortic biopsies were obtained from non-pathological aortas of postmortem heart-beating kidney donors, taken from the abdominal aorta at the level of the renal artery origin. SMC proteomics analysis was performed on smooth muscle cells cultured from a total of 32 aortic biopsies (controls n=8; ND-AAA n=19; D-AAA n=5). Key findings were further validated in aortic tissue using proteomics on additional samples (controls n=17; ND-AAA n=42; D-AAA n=15). No matching criteria were applied. The following patient characteristics were reported: age at the time of biopsy, sex, aneurysm size (mm), rupture, hypertension, previous vascular surgery, renal dysfunction and body mass index (BMI). Since the kidney donors remained anonymous, their only reported clinical characteristics were age and sex. For the qPCR experiments evaluating the effect of metformin, SMCs were obtained from six AAA patients included in the SMC proteomics analysis and nine additional AAA patients. For other experiments, there was no overlap between the AAA SMCs used and those included in the SMC proteomics analysis. For this study, blood samples from 12 ND-AAA patients and 10 D-AAA patients were obtained from the biobank and analyzed. All patient material was collected according to the Declaration of Helsinki regulations and the institutional guidelines of the Medical Ethical Committee of Amsterdam UMC, location VU Medical Center. Biobank material release was approved by the Biobank Review Committee of VUmc (TcB VUmc) under approval code 2017.121 [U2019.031].

Aortic biopsy and Cell Culture

Right after the surgeon removed the aortic tissue from the area of maximal dilation during the operation, the sample was immediately placed in NaCl solution and kept at 4°C until it was transported on ice to the laboratory. A part of the intact aortic tissue was sectioned and placed in an aluminum cryo tube, flash-frozen in liquid nitrogen, and stored at −80 °C until further processing. From the remaining aortic tissue, the intima and adventitia layers were removed using a scalpel. Small pieces, roughly 1 mm2, of the media layer containing SMCs, were dissected and placed in a 25 cm^2 culture flask with 1.5 ml of M231 (Medium 231, Smooth Muscle Cell medium, Gibco, Life Technologies, Carlsbad, CA, USA) supplemented with Penicillin and Streptomycin (10.000 U/mL Penicillin and 10.000 µgr/mL Streptomycin (100x stock), with a final concentration in medium of 100 U/mL penicillin 100 µgr/mL Streptomycin)(Gibco, Life Technologies) and 5% Smooth Muscle Growth Supplement (SMGS, Gibco, Life Technologies). SMCs were placed in a humidified incubator at 37°C, 5% CO2. The culture medium was replaced twice a week until the cells became subconfluent. Subsequently, cells were transferred into a culture flask of 75 cm^2. A confluent population of cells was established approximately six weeks after the day the initial aortic biopsy was collected. Primary SMCs were used between passages one to nine in all experiments.

Plasma Samples

Before the incision, blood samples were collected in 6 mL EDTA tubes and transported to the laboratory at room temperature (RT). Sample were centrifuged at 2000 x *g* for 10 minutes at RT, after which the plasma was collected and stored at -80°C.

Proteomics Analysis

SMC Proteomics Analysis

SMC Sample preparation

Although, there was no power calculation done for the sample size for ND-AAA-SMC and D-AAA-SMC as two separate study groups, a power calculation to determine the sample size for the aortic controls and AAA patients was performed for our previous study(13). In a pilot study (K.B. Rombouts and K.K. Yeung, unpublished data, 2022), a ≈3.5-fold difference in protein expression between aortic SMCs from controls (1±0.42) and AAA patients (3.51±2.20) was found. Additionally, in previous studies, we found more variation in findings in the AAA-SMC group compared with more consistent findings in the C-SMC group, so a higher number of AAA-SMCs compared with C-SMCs should be included. Accordingly, with a power of 80%, α of 0.05, and enrollment ratio of 3:1, we need to use 24 AAA-SMCs and 8 C-SMCs. Every SMC line was cultured in 15-cm dishes until 70-80% confluency was reached. After washing twice with cold PBS, cells were lysed (2 mL lysis buffer for 3 dishes) and detached using a cell scraper. Lysis buffer consisted of 20 mmol/L HEPES pH 8.0, 9 M urea (CH4N2O), 1 mmol/L orthovanadate (Na3VO4), 2.5 mmol/L NaPPi (Na4P2O7 [pyrophosphate]), and 1 mmol/L β-glycerophosphate (Na2C3H7PO6) in MilliQ. As phosphorylation is a rapidly changing state of proteins, the complete procedure was performed within 1 minute. Lysates were sonicated on ice, and debris was removed by centrifugation for 15 minutes at 5400g at 17 °C. Protein concentration was measured using Pierce BCA Protein Assay Kit (Thermo Fisher Scientific, Waltham, MA, USA). For quality control, samples were loaded on a precast 4% to 12% NuPAGE Novex Bis-Tris 1.5-mm mini gel (Invitrogen, Waltham, MA, USA). Electrophoresis was performed at 200 V in NuPAGE MES SDS running buffer until the dye reached the bottom of the gel. Subsequently, gels were fixed in 50% ethanol and 3% phosphoric acid solution and stained with 0.1% coomassie brilliant blue G-250 solution (34% methanol, 3% phosphoric acid, and 15% ammonium sulfate; Figure S1). Lysates were stored in −80 °C until further use. Lysates were thawed, and insoluble material was removed by centrifugation. Samples were reduced with dithiotreitol (4mmol/L, 30minutes at 55°C) and alkylated with iodoacetamide (10mmol/L, 15minutes in the dark). Next, the solution was diluted to 2mol/L urea by the addition of 20mmol/L HEPES pH8.0 and digested with sequencing-grade modified trypsin (Promega) at a final concentration of 5μg/mL overnight at RT. Digests were acidified with trifluoroacetic acid (TFA) to a final concentration of 0.1% and desalted using Oasis HLB cartridges (500mg sorbent; Waters) after equilibration in 0.1% TFA. Bound peptides were washed twice with 0.1% TFA, eluted in 80% acetonitrile/0.1% TFA, and lyophilized.

SMC Proteomics Analysis

Peptide digests were dissolved in 20 µL 0.5% TFA/4% acetonitrile before injection; 5 µL was injected using partial loop injection. Peptides were separated using an Ultimate 3000 nano-LC-MS/MS system (Dionex LC-Packings, Amsterdam, the Netherlands) equipped with a 50-cm, 75-mm ID C18 Acclaim pepmap column (Thermo Scientific). After injection, peptides were trapped at 3 mL/min on a 10-mm, 75-mm ID Acclaim Pepmap trap column (Thermo Scientific) in buffer A (0.1% formic acid) and separated at 300 mL/min with a 10% to 40% buffer B (80% acetonitrile/0.1% formic acid) gradient in 90 minutes (120-minute inject to inject). Eluting peptides were ionized at +2 kV and introduced into a Q Exactive HF mass spectrometer (Thermo Fisher, Bremen, Germany). Intact masses were measured in the Orbitrap cell with a resolution of 120,000 (at m/z 200) using an automatic gain control target value of 3×10^6 charges. The top 15 highest signal peptides (charge states ≥2+) were submitted to MS/MS in the higher energy collision cell (1.6-Da isolation width, 25% normalised collision energy). MS/MS spectra were measured in the Orbitrap with a resolution of 15,000 (at m/z 200) using an automatic gain control target value of 1×10^6 charges and an underfill ratio of 0.1%. Dynamic exclusion was used with a repeat count of 1 and an exclusion time of 30s.

SMC Protein Quantification

MS/MS spectra were searched against a Swissprot reference proteome (human, 2021_01 canonical plus isoforms, 42,383 entries) using MaxQuant 1.6.10.43. Enzyme specificity was set to trypsin, and up to 2 missed cleavages were allowed. Cysteine carboxamidomethylation (+57.021464 Da) was treated as a fixed modification and methionine oxidation (+15.994915 Da) and N-terminal acetylation (+42.010565 Da) as variable modifications. Peptide precursor ions were searched with a maximum mass deviation of 4.5 ppm and fragment ions with a maximum mass deviation of 20 ppm. Peptide, protein, and site identifications were

filtered at a false discovery rate of 1% using the decoy database strategy. The minimum peptide length was set at 7 amino acids, the minimum Andromeda score for modified peptides was 40, and the corresponding minimum delta score was 6 (default MaxQuant settings).

SMC Proteomics Data Analysis

Protein-level differential analyses were performed using spectral count data. Counts were normalized on the total count per sample relative to the average sample total in the dataset. Group differences were tested using a beta-binomial test for the independent samples(14). In addition to a significant *P* value ($P<0.05$), data were filtered to find the most discriminatory changes. Proteomics data were filtered for proteins found in ≥50% of the samples within one or both groups to compare C-SMC and ND-AAA-SMC. Proteomics data were filtered for proteins found in ≥75% of the samples within one or both groups to compare ND-AAA-SMC and D-AAA-SMC. Venn diagrams were conducted using jvenn(15). KEGG and Reactome pathway, Gene Ontology (GO) analyses were performed using ShinyGO 0.81(16, 17), with the complete set of identified proteins serving as the background for the KEGG and GO analyses. Results were filtered based on a False Discovery Rate (FDR) threshold, and only terms with at least two proteins were included. KEGG pathways were selected based on their relevance to the objectives of this study. Overlapping GO terms involving the same protein group were reduced by selecting a single representative term. Visualisations were generated using SR Plot(18). Protein-protein interaction (PPI) analysis was performed using the Search Tool for the Retrieval of Interacting Genes/Proteins database (String version 12.0) and visualised with Cytoscape software (version 3.10.3)(19, 20). Hub proteins were identified using the CytoHubba program, with the Maximal Clique Centrality (MCC) method(21). Other Graphs were constructed using GraphPad Prism 10.2.0 (GraphPad Software, San Diego, CA, USA) and SR plot and results were expressed as median with interquartile range.

Data Availability

The mass spectrometry proteomics data have been deposited to the ProteomeXchange consortium via the PRIDE (Proteomics Identifications)(22) partner repository with the data set identifier PXD054353.

Tissue Proteomics analysis

To validate SMC proteomics findings in the intact aortic wall, protein expression of selected proteins from the identified PPI networks were analyzed in aortic tissue using a proteomics approach.

Tissue Sample Preparation

Tissue homogenization was performed using a bead-beating system (TissueLyser II, QIAGEN, Hilden, Germany) with zirconium dioxide beads (Bertin Technologies, Montigny-le-Bretonneux, France). The homogenization protocol was carried out under the following conditions: (a) bead diameter of 1.4 mm; (b) three extraction cycles, each defined as a single homogenization event followed by centrifugation; (c) a bead mass-to-tissue mass ratio of 30:1; and (d) a lysis buffer volume-to-tissue mass ratio of 20 µL/mg. Each homogenization cycle was conducted for 30 seconds, with a 5-minute cooling interval on ice between cycles. Lysis was performed using RIPA buffer (25 mM Tris-HCl pH 7.6, 150 mM NaCl, 1% NP-40, 1% sodium deoxycholate, 0.1% SDS), prepared in-house and supplemented with 1 mM EDTA (EDTA disodium salt dihydrate, ≥99%, Merck-Millipore, Darmstadt, Germany), a protease inhibitor cocktail (Roche Diagnostics GmbH, Mannheim, Germany), and phosphatase inhibitors (1 mM sodium orthovanadate (Na_3VO_4), 2.5 mM sodium pyrophosphate ($Na_4P_2O_7$), and 1 mM β-glycerophosphate). Following homogenization, lysates were centrifuged at 18,626 × g for 30 minutes at 4 °C (Mikro 200 R centrifuge, Hettich Zentrifugen, Tuttlingen, Germany). The resulting protein-rich supernatants were collected and stored at −80 °C until further processing. Protein concentrations were determined using the Detergent-Compatible (DC) Protein Assay Kit (Bio-Rad Laboratories, Hercules, CA, USA), with bovine serum albumin as the standard. Samples were normalized to contain 10 µg of total protein in 50 µL of lysate for subsequent Suspension-Trapping (S-Trap) sample processing (see below).

2.2.2.2 Tissue Proteomics Analysis

For proteomics analysis, lysates were subjected to a manual S-Trap procedure(23, 24). This included reduction and alkylation of proteins in the high-detergent lysate and further denaturation mediated by 1% phosphoric acid and 90% methanol to give a colloidal protein suspension that is trapped in DNA miniprep spin filters. Trapped proteins were digested on-filter with sequencing-grade modified trypsin (1:10 m/m) (Promega, Leiden, Netherlands) and tryptic peptides were recovered by successive extractions with 0.1% formic acid and 0.1% formic acid/acetonitrile. After vacuum centrifugation to dryness and resollubilisation in 0.1% FA, 400 ng aliquots were loaded on Evotips according to the manufacturer's instructions (Evosep, Denmark). Peptides were separated via nanoflow reversed-phase liquid chromatography using an Evosep One liquid chromatography system (Evosep, Denmark) with 0.1% FA and 0.1% FA/99.9% ACN as the mobile phases A and B, respectively. The 30 samples per day (30SPD) pre-set Evosep method was combined with a 15 cm x 150 µm ID PepSep reversed-phase column packed with 1.5 µm C18-beads (Bruker Daltonics) resulting in a 44 min. inject-to-inject time. 50 ng HeLa digests (Pierce Thermo) were used as QC samples after every 10 plasma injections. The nanoLC column was connected to a 20 µm ID fused silica emitter (Bruker Daltonics) in the Captive spray source (Bruker Daltonics) operating at a spray voltage of 1400 V. Peptides were analyzed with a TimsTOF HT (Bruker Daltonics) running in DIA-PASEF mode. The DIA-PASEF method was optimized for the specific sample type using the py_diAID tool. The method covered an ion mobility range from 1.5 to 0.7 Vs cm^{-2} and an m/z range of 300 to 1200, using 10 DIA-PASEF scans with two isolation windows per scan, resulting in a cycle time of 1.1 s. Collision energy was linearly decreased from 59 eV at 1.6 Vs cm^{-2} to 20 eV at 0.6 Vs cm^{-2}. For spectral library construction data was acquired in DDA-PASEF mode optimized for spectral quality (long IM ramp). The ramp time was set to 200 ms and ten PASEF scans were acquired per topN acquisition cycle, resulting in a cycle time of 2.16s. Precursors with a mass range from 100 m/z to 1700 m/z, ion mobility range from 1.5 to 0.7 Vs cm^{-2}, and charge states from 0 (unassigned) to 5+ were analyzed.

<u>2.2.2.3 Tissue Protein Quantification</u>

DIA MS/MS spectra were searched against the Uniprot reviewed human canonical and isoform FASTA file (downloaded 2023-03-30) using DIA-NN v1.9.2(25). Enzyme specificity was set to trypsin and up to two missed cleavages were allowed. Cysteine carboxamidomethylation (+57.021464 Da) was treated as fixed modification and Methionine oxidation (+ +15.994915 Da) as variable modification. Peptide length was set to 7-30 amino acids and the precursor m/z was set to 300-1200. Charge states 2-4+ were considered and the MS1 and MS2 thresholds were set to 10 ppm and data was filtered to a Q-value of 1%. Match-between-runs (MBR), heuristic protein inference and no shared spectra options were selected. Protein inference was based on genes, machine learning was single-pass NNs, quantification strategy was UMS (high accuracy), cross-run normalization was RT dependent, library generation included ID, RT and IM, and speed and RAM usage was optimized for optimal results. The human plasma dataset was analyzed using a project-specific cumulative spectral library based on high-pH reversed phase separated peptides (concatenated fractions) and in-gel digested pooled plasma samples. Data was processed with FragPipe v20.0. to generate a spectral library from the DDA-PASEF data. The human tissue dataset was analyzed using a predicted spectral library based on the FASTA file. DIA-MS detected plasma and tissue peptides were quantified from MS2 signals (area) and aggregated to the protein group level. Protein intensities were calculated using a DIA MaxLFQ algorithm(26) and were normalized per sample to the median intensity across samples and no imputation was applied.

<u>2.2.2.4 Tissue Proteomics Data Analysis</u>

Analysis and statistical testing were performed in R (version 4.4.2). The statistical analysis of patient characteristics did not account for missing data. The Wilcoxon and Fisher's exact tests were used to compare patient characteristics between the two study groups. Tissue proteomics samples with less than 3000 identified proteins were removed. Differences in protein abundances were tested using the Mann-Whitney U test for two-group comparisons, based on log2-transformed intensity values. Differences with a p-value below 0.05 were considered significant. Proteins that were present and quantified in less than 50% in both

examined subpopulations were excluded from further analysis. Graphs were constructed using GraphPad Prism 10.2.0 (GraphPad Software, San Diego, CA, USA).

2.2.1.5 Data Availability

The mass spectrometry proteomics data have been deposited to the ProteomeXchange consortium via the PRIDE (Proteomics Identifications)(26) partner repository with the tissue data set identifier PXD067859.

Experiments To test the Effect of Metformin on SMCs

Metformin Dosage

The concentration of 10 mM metformin hydrochloride (Toronto Research Chemicals Inc., Toronto, ON, Canada) was used based on literature, as on preliminary results of our previous study in which we determined that this concentration is not harmful for the cells(8).

RNA isolation and Quantitative Polymerase Chain Reaction

To test the effect of metformin on cytoskeleton, ECM, mitochondrial markers and genes involved in the reaction against redox stress in SMCs, mRNA expression levels of specific genes, were measured. The plates were coated with 0.1% gelatin for 30 minutes (37°C). SMCs were seeded at a density of 200 000 cells per well in two wells of a 6-well plate with 2 ml supplemented M231. After incubating overnight to allow cell attachment and monolayer formation, one well per cell line was treated with supplemented M231, while the other received supplemented M231 with 10 mM metformin. The medium was refreshed after three days. Following five days of treatment, cells were washed once with PBS (37°C), and SMCs were homogenized individually in 300 µL of lysis buffer (Zymo Research, Irvine, CA, USA) in 2.0 mL Eppendorf tubes. Total RNA was isolated using the Quick-RNA™ MiniPrep kit (Zymo Research) following the manufacturer's instructions. After RNA isolation, RNA purity was assessed using the NanoDrop™ One Spectrophotometer (Fisher Scientific, Loughborough, Leicestershire, UK) based on the A260/A280 ratio. Samples with A260/A280 ≥ 1.8 were considered suitable for complementary DNA synthesis. First-strand complementary DNA was synthesized in a 20 µL reverse transcription reaction using a VILO kit (Thermo Fisher Scientific), with adjustments made according to the concentration of isolated RNA. Real-time quantitative Polymerase Chain Reaction (RT-qPCR) was conducted with iQ SYBR Green Supermix (Bio-RAD, Hercules, CA, USA) on the Bio-Rad CFX384 Touch Real-time PCR Detection System, and mRNA levels of target genes were normalized to the housekeeping gene TATA-box binding protein (TBP). Gene expression analysis was performed using the $2^{-\Delta CT}$ method. Primer sequences are provided in Table S1.

Western blotting

The plates were coated with 0.1% gelatin for 30 minutes (37°C). SMCs were seeded at a density of 100 000 cells per well in two wells of a 6-well plate with 2 ml supplemented M231 medium. The cells were given two days to attach and form a monolayer. Subsequently, metformin was added to the existing medium of one well per cell line, resulting in a final concentration of 10 mM. After six hours of treatment, SMCs were washed with PBS and subsequently lysed in 130 µl SDS sample buffer (containing 125 mM Tris-HCl pH 6.8, 4% SDS, 20% glycerol, 100 mM DTT, 0.02% Bromophenol Blue in MilliQ). The protein concentration was measured with a Pierce BCA Protein Assay Kit (Thermo Fisher Scientific, Waltham, MA, USA) according to the manufacturer's instructions, to ensure equal protein loading across samples Following a 10-minute boiling step at 95 °C, 15 µl of each sample was loaded onto SDS-PAGE gels for protein separation. Nitrocellulose membranes were then used for protein transfer. The membranes were blocked by incubating with BSA 5% for 1h at RT on a rocking platform. Primary antibodies against Phospho-AMPKα(Thr172)(1:1000; Cell signaling Technology, #2535), AMPKα (1:1000; Cell Signaling Technology; #2603), KEAP1 (1:1500; Proteintech (Proteintech Group Inc., Rosemont, IL, USA), #10503-2-AP) and β-Actin (1:1000; Cell Signaling Technology, #4967) as loading control were incubated overnight at 4°C. Secondary antibody incubation was performed for 1 hour at RT with HRP conjugated polyclonal goat anti-rabbit immunoglobulin (1:5000;

Dako(Carpinteria, CA, USA), #P0448), diluted in milk powder. Proteins were visualized with enhanced chemiluminescence (GE Healthcare, Little Chalfont, Buckinghamshire, UK) using the Amersham Imager 600 (GE Healthcare). Analysis of the band intensities was performed using ImageQuantTL.

Immunofluoresence

The SMCs were cultured in 96 well cell culture microplates with F-bottom (Greiner Bio-One, Alphen aan den Rijn, South Holland, Netherlands, #655090). The plates were coated with 0.1% gelatin for 30 minutes (37°C). In the 96 well plate, SMCs were seeded in eight wells per cell line in a density of 20 000-30 000 cells/well in a cell suspension of 200 µl in complete cell culture medium. The cells were given two days to attach and form a monolayer. Four wells per cell line were treated with supplemented M231 and the other four with supplemented M231 containing 10 mM metformin. Cells were fixed and stained for NAD(P)H Quinone Dehydrogenase 1 (NQO1) and Nuclear Factor Erythroid 2-Related Factor 2 (NRF2) after four days and three days respectively. Cells were washed with PBS and fixed with 4% PFA in PBS for 10 minutes. They were then washed three times with PBS for five minutes on a shaker at RT, followed by three washes with IF buffer-0.3% BSA (containing 20% Triton, 20% Tween, and 0.3% BSA in PBS) under the same conditions. Blocking was performed with IF buffer-3% BSA for one hour on a shaker at 4°C. Primary antibodies diluted in IF buffer-3% BSA and were added to four wells per cell line (two untreated and two metformin-treated) for 24 hour on a shaker at 4°C: anti-phospho-Nrf2 Ser40 (1:250; Abcam, #ab76026) and NQO1 (1:150; Cell Signaling, #62262). Afterwards, the cells were washed three times with IF buffer-0.3% BSA for five minutes on a shaker at RT. Donkey anti-Rabbit IgG (H+L) Highly Cross-Adsorbed Secondary Antibody, Alexa Fluor™ 488 (Thermo Fisher Scientific #A-21206) and Acti-Stain 670 Phalloidin (Cytoskeleton Inc., #PHDN1) were both diluted 1:250 in IF buffer-3% BSA and added to the cells overnight on a shaker at 4°C. The cells were washed twice with IF buffer-0.3% BSA for five minutes on a shaker at RT. DAPI (4′,6-diamidino-2-phenylindole) (1:1000, Thermo Fisher Scientific, #62248) was diluted in IF buffer-3% BSA and added to the wells for 10 minutes on a shaker at 4°C. Cells were then washed twice with PBS for five minutes on a shaker at RT and stored at 4°C until imaging. Images were captured using an ImageXpress Pico Automated Cell Imaging System (Molecular Devices, San Jose, CA, USA) and were analyzed using the associated software. Confocal z-stacked images were made using the 60× objective on the spinning disk microscope (Nikon Eclipse Ti2, Nikon Corporation, Tokyo, Japan) and visualized using ImageJ 1.49 (National Institutes of Health, Bethesda, MD, USA).

Pentosidine Measurement

The ELISA kit for Pentosidine (CLOUD-CLONE CORP, Houston, TX, United States, #CEA264Ge) was used to measure the advanced glycation end products (AGEs) generation in cell culture supernatants. SMCs were seeded in 2 wells per cell line in a gelatine-coated 6-well plate at a density of 200 000 cells/well in a cell suspension of 2000 µl in supplemented M231. Cells were incubated overnight, allowing cells to attach and establish a monolayer. One well per cell line was treated with supplemented M231 and the other with supplemented M231 containing 10 mM metformin. The cell culture supernatants were collected after three days. The ELISA kit for Pentosidine was used according to the manufacturer's protocol.

Baseline Measurement of Oxygen Consumption and Extracellular Acidification

Mitochondrial oxygen consumption rate (OCR) and Extracellular Acidification Rate (ECAR) were measured using the Seahorse XFe96 Analyzer (Agilent). SMCs were seeded in six wells per cell line in a gelatine-coated 96-well plate at a density of 20 000 cells/well in a cell suspension of 80 µl in supplemented M231. The plate was left at RT for one hour to allow the cells to attach before being moved to the incubator overnight. Three wells per cell line were treated with supplemented M231 and the other three were treated with supplemented M231 containing 10 mM metformin. After 24 hours, the SMCs were washed with 180 µl pre-warmed complete Seahorse Assay Medium (DMEM base medium supplemented with 1 mM pyruvate, 2 mM glutamine, 10 mM glucose, 5 mM HEPES and 1.3 mM bicarbonate). After, 180 µL of the complete Seahorse Assay medium was added and the cells were incubated in a 37 °C non-CO2 incubator for 45 mins prior to

assay. The plate was loaded in the Bioanalyzer and the OCR and ECAR were measured. The values were normalized by counting cell nuclei using Hoechst 33342(1:1000, Thermo Fisher Scientific, #62249).

L-lactate Assay

To measure anaerobic glycolysis, an L-lactate assay was used to determine the amount of lactate secreted by SMCs. SMCs were seeded in 2 wells per cell line in a gelatine-coated 6-well plate at a density of 200 000 cells/well in a cell suspension of 2000 µl in supplemented M231. Cells were incubated overnight, allowing cells to attach and establish a monolayer. One well per cell line was treated with supplemented M231 and the other with supplemented M231 containing 10 mM metformin. The cell culture supernants were collected after three days. The samples were deproteinized with 5% metaphosphoric acid (MPA) (Sigma-Aldrich ,St. Louis, MO, USA; 239275). A lactate standard was prepared for comparison. A master mix containing 0.5 M glycine-0.4 M hydrazine buffer (pH 9.0) and 27 mM nicotinamide adenine dinucleotide (NAD) was prepared. Additionally, a start solution was made, containing 0.5 M glycine-0.4 M hydrazine buffer and L-lactate dehydrogenase (5 mg/ml) (Roche, Basel, Switzerland). The master mix was pipetted into all wells of a 96-well plate, followed by the deproteinized samples or lactate standard. To normalize the final results, NADH fluorescence was measured using a NOVOStar (BMG Labtech, Ortenberg, Germany) with λex/λem = 340-10/450-10 nm every 2 minutes for 5 cycles. The start solution was added to the wells, and NADH fluorescence was measured similarly every 2 minutes, this time with shaking, until a stable reading was obtained.

NQO1 Activity Assay Kit

The NQO1 Activity Assay kit (Abcam, Cambridge, United Kingdom, #ab184867) was used according to the manufacturer's protocol to determine the NQO1 enzymatic activity as proxy for the amount of NQO1 protein levels in plasma.

Statistics

Statistical analyses were conducted using SPSS (version 28, IBM Statistics). Both ANOVA and χ^2 tests were used to evaluate clinical characteristics across three study groups. The independent samples t-test and χ^2 test were utilized to compare patient characteristics between two study groups. The statistical analysis of patient characteristics did not account for missing data. When datasets were normally distributed, either the paired t-test or the independent samples t-test was used. A two-sided p-value is reported, except when a one-sided test was applied due to a specific directional hypothesis, and this is explicitly stated in the text. When datasets were not normally distributed, nonparametric tests were applied. The Mann-Whitney U test was used to compare two independent groups. The Wilcoxon Signed-Ranks Test was performed to assess differences between related groups (the untreated and metformin-treated groups). Results are shown in plots with the mean and standard deviation (SD) for parametric tests, while plots representing the median and interquartile range (IQR) are used for nonparametric tests. Statistical significance was defined as $p<0.05$. Plots were generated using GraphPad Prism version 10.2.0.

Major Resources Table

Antibodies

Target antigen	Vendor or Source	Catalog #	Working concentration	Lot # (preferred but not required)	Persistent ID / URL
Phospho-AMPKα(Thr172)	Cell Signaling Technology	2535	1:1000		Phospho-AMPKα (Thr172) (40H9) Rabbit mAb | Cell Signaling Technology
AMPKα	Cell Signaling Technology	2603	1:1000		AMPKα (23A3) Rabbit mAb | Cell Signaling Technology
KEAP1	Proteintech	10503-2-AP	1:1500		KEAP1 antibody (10503-2-AP) | Proteintech
β-Actin	Cell Signaling Technology	4967	1:1000		β-Actin Antibody | Cell Signaling Technology
HRP conjugated polyclonal goat anti-rabbit immunoglobulin	Dako	P0448	1:5000		Goat Anti-Rabbit Ig/HRP Antibody (affinity isolated) | Agilent
Acti-Stain 670 Phalloidin	Cytoskeleton Inc	PHDN1	1:250		Acti-stain 670 phalloidin - Cytoskeleton, Inc.
DAPI	Thermo Fisher Scientific	62248	1:1000		DAPI and Hoechst Nucleic Acid Stains
anti-phospho-Nrf2 Ser40	Abcam	ab76026	1:250		Anti-phospho NRF2 (pS40) antibody [EP1809Y] - recombinant (ab76026) | Abcam
NQO1	Cell Signaling Technology	62262	1:150		NQO1 (D6H3A) Rabbit mAb | Cell Signaling Technology
Hoechst 33342	Thermo Fisher Scientific	62249	1:1000		DAPI and Hoechst Nucleic Acid Stains

Cultured Cells

Name	Vendor or Source	Sex (F, M, or unknown)	Persistent ID / URL
Primary SMC from healthy aortic biopsies (C-SMC)	Non-dilated infrarenal heart-beating kidney donors (without CVD)	Both F and M (see Table 1)	
Primary SMC from AAA aortic biopsies (AAA-SMC)	The area of maximal dilation of the aorta of AAA patients	Both F and M (see Table 1)	

Other

Description	Source / Repository	Persistent ID / URL
96 well cell culture microplates with F-bottom	Greiner Bio-One, #655090	CELLSTAR® plaat, 96w, F, µClear®, schoorsteen, - 655090
ELISA kit for Pentosidine	CLOUD-CLONE CORP, #CEA264Ge	ELISA Kit for Pentosidine (PTD) | CEA264Ge | Pan-species (General) CLOUD-CLONE CORP.(CCC)
L-Lactate assay	Roche, #MAK329	L-Lactate Assay Kit, MAK329, 100 Tests, Sigma-Aldrich
NQO1 Activity Assay kit	Abcam, #ab184867	NQO1 Activity Assay Kit (ab184867) | Abcam

Supplemental Figures

Figure S1. Coomassie blue staining of samples for quality control

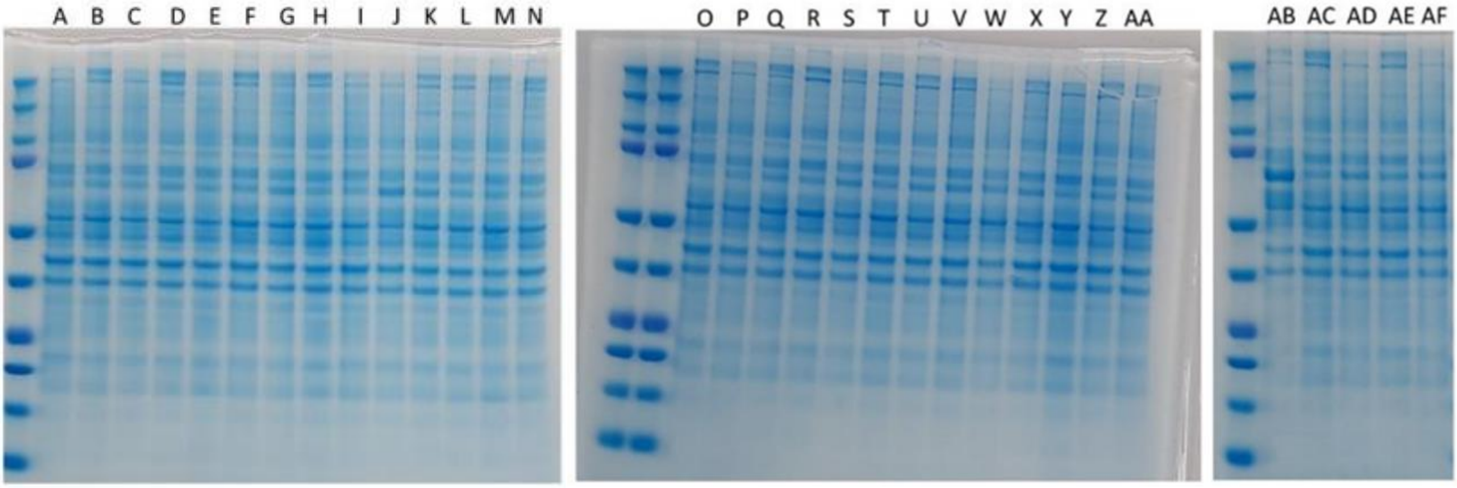

Slot	Letter	Group	µg loaded
1	Ladder		
2	A	ND-AAA	9 µg
3	B	ND-AAA	9 µg
4	C	ND-AAA	9 µg
5	D	Control	9 µg
6	E	ND-AAA	9 µg
7	F	ND-AAA	9 µg
8	G	ND-AAA	9 µg
9	H	Control	9 µg
10	I	ND-AAA	9 µg
11	J	ND-AAA	9 µg
12	K	ND-AAA	9 µg
13	L	Control	9 µg
14	M	D-AAA	9 µg
15	N	ND-AAA	9 µg

Slot	Letter	Group	µg loaded
1	Ladder		
2	Ladder		
3	O	D-AAA	9 µg
4	P	Control	9 µg
5	Q	ND-AAA	9 µg
6	R	ND-AAA	9 µg
7	S	ND-AAA	9 µg
8	T	Control	9 µg
9	U	ND-AAA	9 µg
10	V	ND-AAA	9 µg
11	W	D-AAA	9 µg
12	X	Control	9 µg
13	Y	ND-AAA	9 µg
14	Z	D-AAA	9 µg
15	AA	Control	9 µg

Slot	Letter	Group	µg loaded
1	Ladder		
2	AB	D-AAA	9 µg
3	AC	ND-AAA	9 µg
4	AD	Control	9 µg
5	AE	ND-AAA	9 µg
6	AF	ND-AAA	9 µg

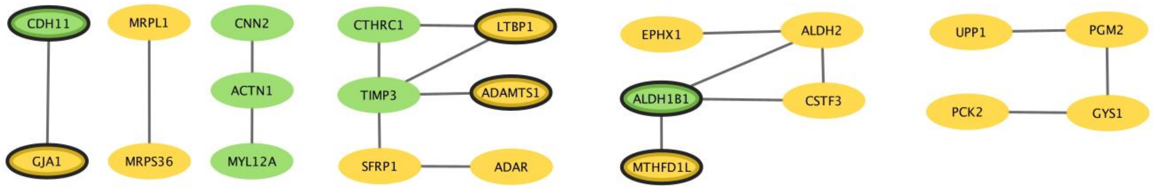

Figure S2. Protein-protein interaction (PPI) network analyses performed for the proteins with increased and decreased expression in ND-AAA-SMC compared to C-SMC. Proteins with lower expression in ND-AAA-SMCs highlighted in yellow, and proteins with higher expression in ND-AAA-SMCs compared to C-SMCs highlighted in green. Proteins that are part of the cluster, since the proteins with increased and decreased expression were taken together, are highlighted with a thick outline.

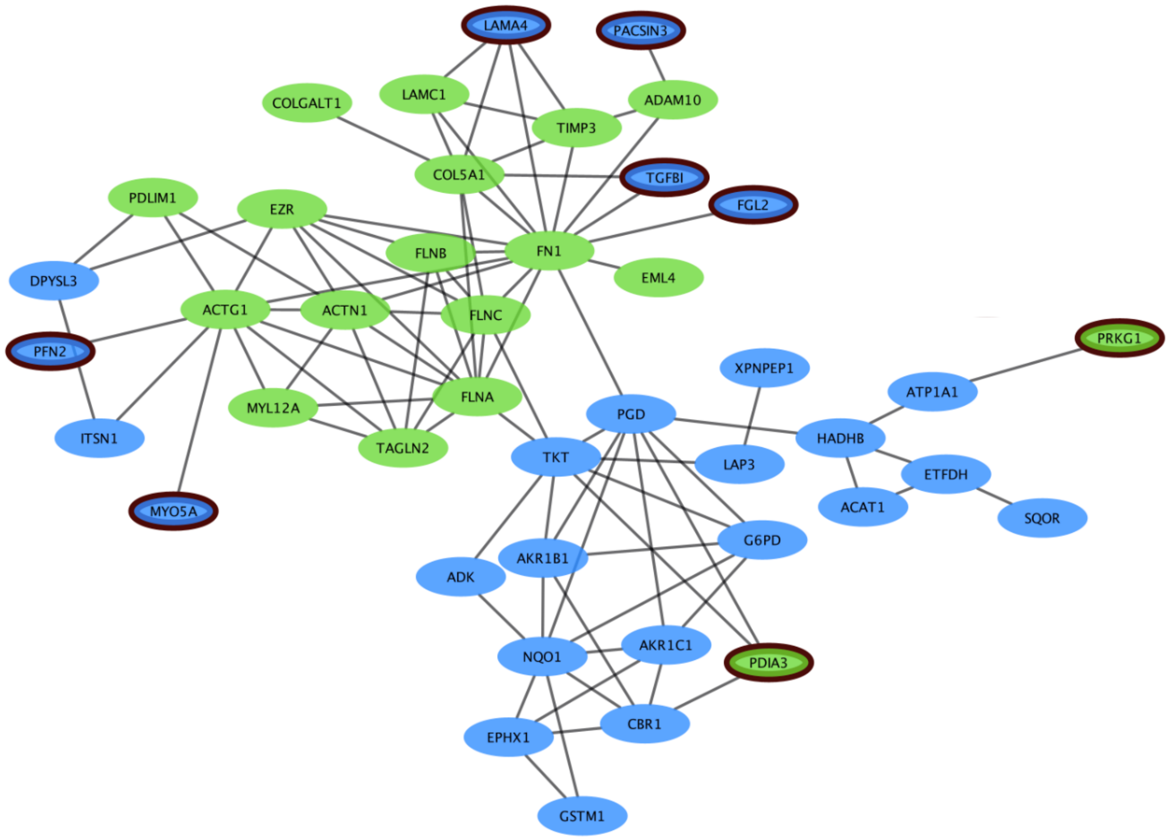

Figure S3. Protein-Protein Interaction (PPI) network analyses performed for the proteins with increased and decreased expression in D-AAA-SMC compared to ND-AAA-SMC. Proteins with lower expression in D-AAA-SMCs highlighted in green, and proteins with higher expression in D-AAA-SMCs compared to ND-AAA-SMCs highlighted in blue. Proteins that have now become part of the integrated cluster of the proteins with increased and decreased expression together, are highlighted with a thick outline.

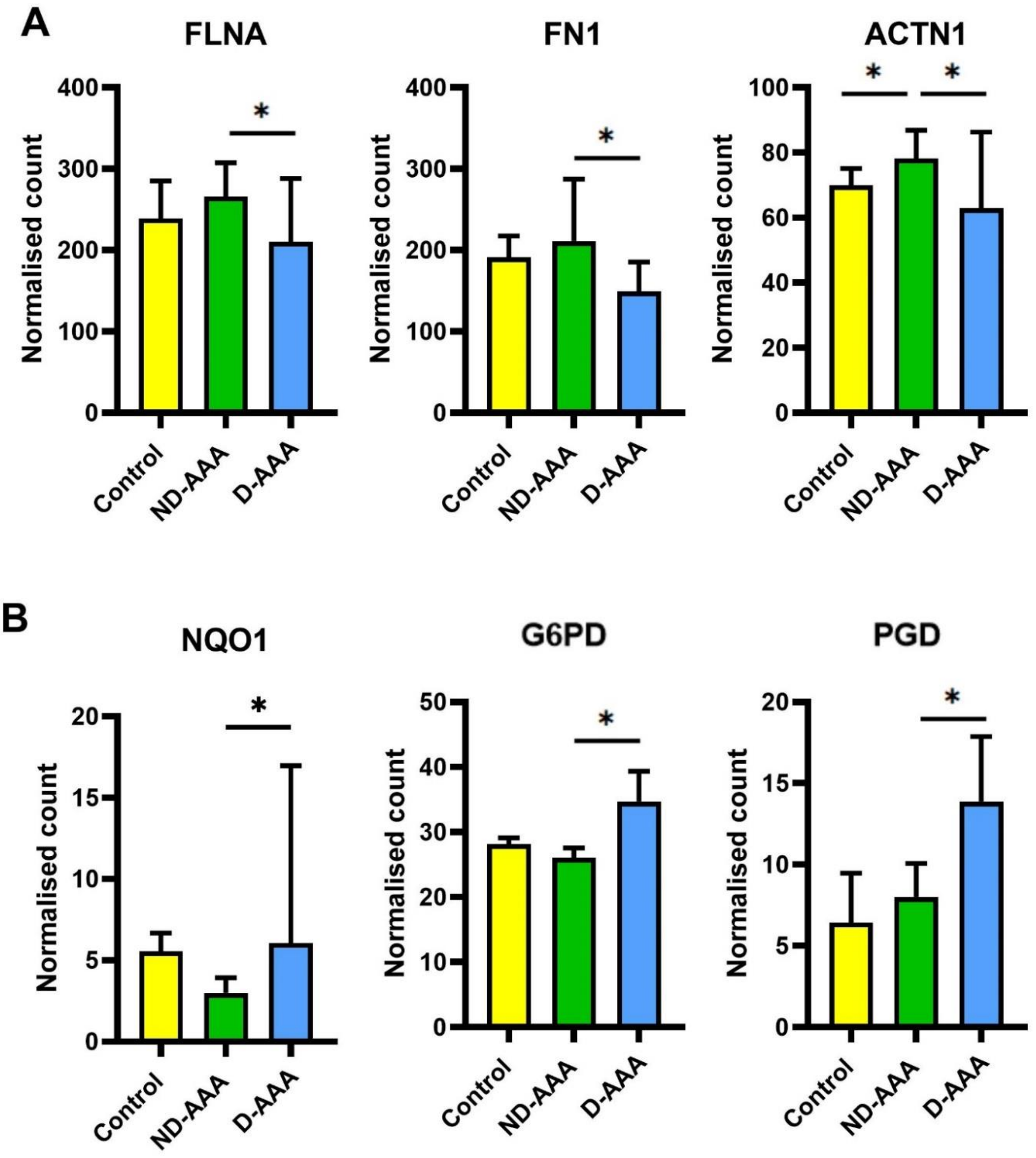

Figure S4. Expression of hub proteins in aortic SMC derived from diabetic AAA patients compared to non-diabetic AAA patients. A. Protein expression of the proteins that were decreased in D-AAA-SMCs compared to ND-AAA-SMCs. Filamin A (FLNA)(log2 (Fold Change(FC)) =-1.17, *p*=0.025), Fibronectin 1 (FN1)(log2(FC)=-1.46, *p*=0.036), and Actinin Alpha 1 (ACTN1)(log2(FC)=-1.20, *p*=0.032). In addition, there was a significant difference comparing the expression of ACTN1 between SMCs of controls (C) and ND-AAA patients (log2(FC)=1.13, *p*=0.028). **B.** Protein expression of the proteins that were increased in D-AAA-SMCs compared to ND-AAA-SMCs. NAD(P)H: Quinone Oxidoreductase 1 (NQO1)(log2(FC)=2.94, *p*=0.019), Glucose-6-Phosphate Dehydrogenase (G6PD)(log2(FC)=1.24, *p*=0.026), and 6-Phosphogluconate Dehydrogenase (PGD)(log2(FC)=1.52, *p*=0.047). The counts of the proteomics data shown in A and B were normalized to the total spectral count per sample. The proteomics spectral count data were tested using a β-binomial test for independent samples. Data represent the median and interquartile range, $*$ $p \leq 0.050$; $**$ $p \leq 0.010$.

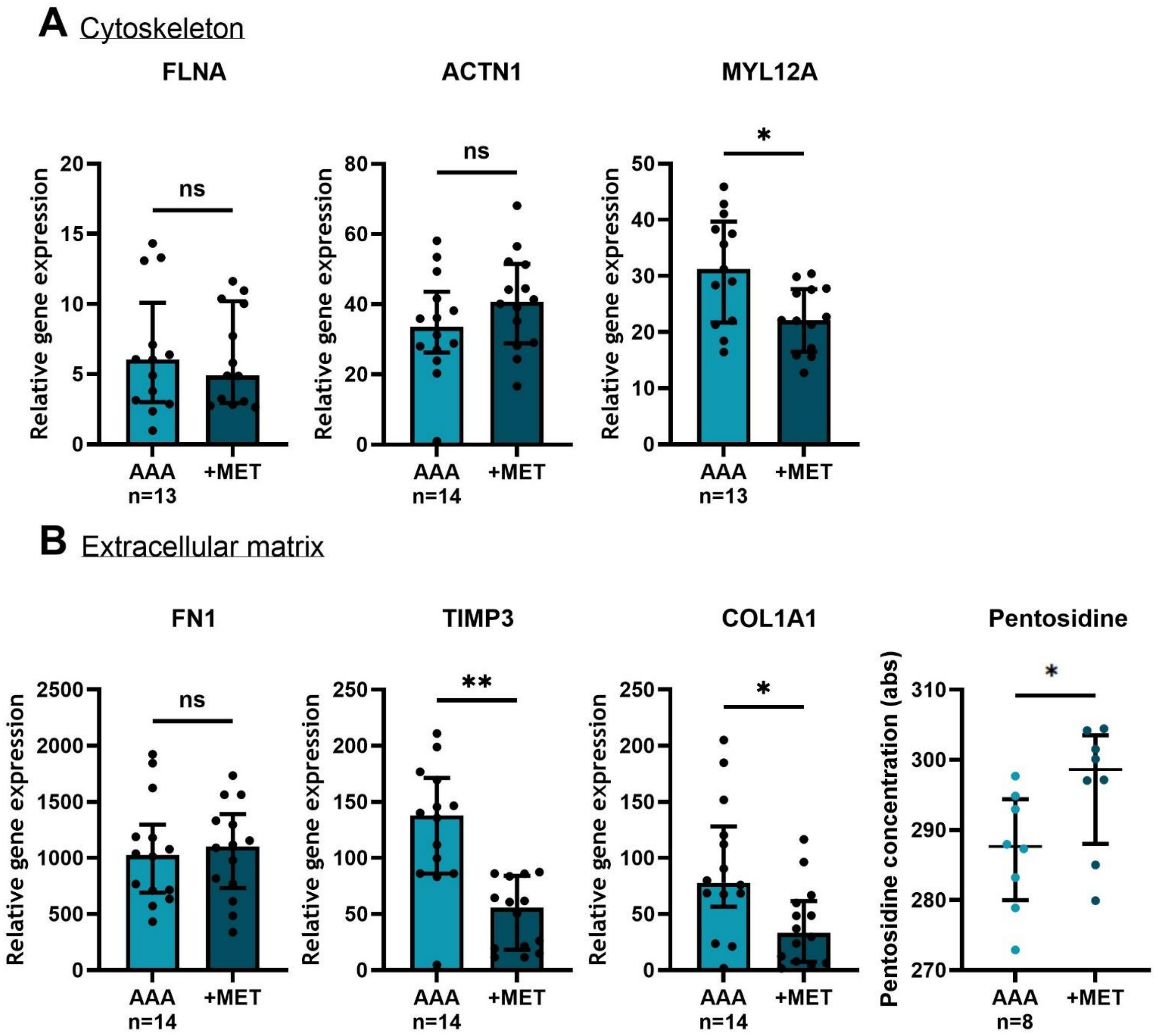

Figure S5. The effect of metformin on the cytoskeleton and extracellular matrix in aortic SMC derived from AAA patients. A. Metformin treatment had no significant effect on the gene expression of Filamin A (*FLNA*) and Actinin Alpha 1 (*ACTN1*)(*p*=0.972 and p=0.245, respectively). However, metformin decreased the gene expression of cytoskeleton marker Myosin Light Chain 12A (*MYL12A*), in SMCs of AAA patients (*p*=0.015). **B.** Metformin treatment had no significant effect on the gene expression of Fibronectin 1 (*FN1*)(*p*=0.975). Moreover, metformin decreased the gene expression of two other extracellular matrix genes, Tissue Inhibitor of Metalloproteinases 3 (*TIMP3*) and Collagen Type I alpha 1 (*COL1A1*)(*p*=0.005 and *p*=0.013, respectively). The formation of Pentosidine, an Advanced Glycation End Product, was increased in cell supernatants after metformin treatment (*p*=0.050). The mRNA levels of target genes were normalized to the housekeeping gene TATA box Binding Protein (*TBP*). The Wilcoxon Signed-Ranks Test was performed to assess differences between the untreated and metformin treated SMCs. Data represent the median and interquartile range. ∗*p*≤0.050; ∗∗*p*≤0.010. MET= metformin.

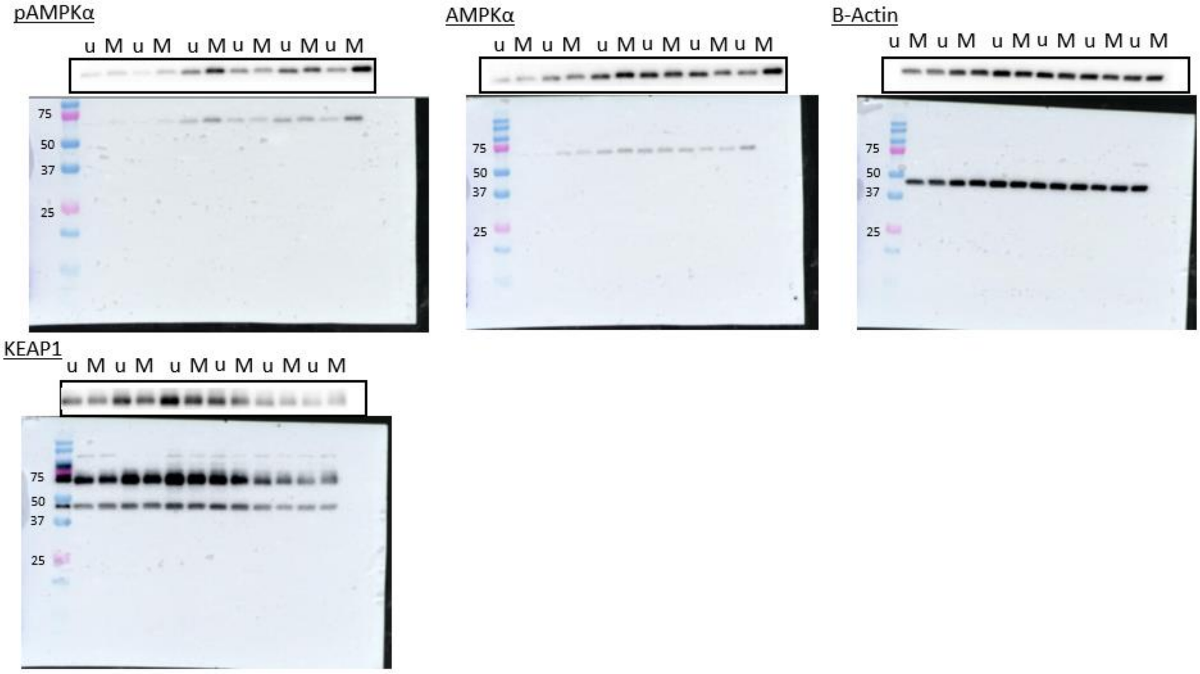

Figure S6. Full unedited western blots. The expression was measured in six different SMC lines derived from AAA patients, untreated (u) and after metformin treatment (M).

Supplemental Tables

Table S1: Primer sequences of genes that were assayed by Quantitative Polymerase Chain Reaction.

**Genes**	**Sequence Forward**	**Sequence Reverse**
FLNA	AAGTGACCGCCAATAACGAC	GGCGTCACCCTGTGACTTAT
ACTN1	GGCAAGATGAGAGTGCACAA	AGATGTCCTGGATGGCAAAG
MYL12A	TCACACGCATCCTGAAACAT	GCCCAAAATGGCTGAGAATA
FN1	ACCAACCTACGGATGACTCG	GCTCATCATCTGGCCATTTT
TIMP3	CTGACAGGTCGCGTCTATGA	GGCGTAGTGTTTGGACTGGT
COL1A1	ATGTCTAGGGTCTAGACATGTTCA	CCTTGCCGTTGTCGCAGACG
PPARA	CTATCATTTGCTGTGGAGATCG	AAGATATCGTCCGGGTGGTT
HADHB	CTGCGGTTTCTGAGTTCTCC	CTGCTCCAGTGAGGAAGGAC
PGC-1a	CCTGCATGAGTGTGTGCTCT	CAGCACACTCGATGTCACTCC
SDHB	GACACCAACCTCAATAAG	GATTCATCCTTCTTCTTCAA
MT-ATP6	TAGCCATACACAACACTAAAGGACGA	GGGCATTTTTAATCTTAGAGCGAAA
G6PD	CTGTTCCGTGAGGACCAGATCT	TGAAGGTGAGGATAACGCAGGC
PGD	ATGCCCTGTTTTACCACTGC	GTCCTGGTCTGTGGAATCGT
CBR1	ATACGGGGTGACGAAGATTG	CACCTTCTTCTGGGCTCTTG
SOD2	TCCACTGCAAGGAACAACAG	TCTTGCTGGGATCATTAGGG
CAT	AGCTTAGCGTTCATCCGTGT	TCCAATCATCCGTCAAAACA
TBP	AGTTCTGGGATTGTACCGCA	TCCTCATGATTACCGCAGCA

Table S2. Clinical characteristics of aortic controls (n=17), non-diabetic (n=42) and diabetic (n=15) abdominal aortic aneurysm patients.

	Control (n = 17)	ND-AAA (n = 42)	D-AAA (n = 15)*	*p* value C vs. ND-AAA vs. D-AAA	*p* value ND-AAA vs. D-AAA
Age (years)	55.9 ± 18.4	73.0 ± 8.0	71.0 ± 7.7	<0.001	0.418
Male	4 (36.4)	31 (75.6)	13 (86.7)	0.013	0.481
Aneurysm size (mm)	N/A	66.0 ± 16.0	66.9 ± 17.7	N/A	0.853
Current smoking	N/A	13 (31.0)	6 (40.0)	N/A	0.539
Hypertension†	N/A	28 (68.3)	13 (86.7)	N/A	0.306
Previous vascular surgery ‡	N/A	12 (29.3)	10 (66.7)	N/A	0.015
Renal dysfunction	N/A	12 (29.3)	5 (33.3)	N/A	0.755
BMI	N/A	26.5 ± 3.5	26.7 ± 4.6	N/A	0.883

Data are presented as *n* (%) or mean ± standard deviation. Valid percentages were reported in cases of missing clinical data. AAA = abdominal aortic aneurysm; ND = non-diabetic; D = diabetic; BMI = Body Mass Index; N/A = not applicable.

*Eight diabetic AAA patients used metformin. Of the seven non-metformin D-AAA patients not using metformin, three were not on antidiabetic medication, two used dapagliflozin, one used tolbutamide, and one used insulin.

†Hypertension and renal dysfunction were defined as diagnosed by a medical doctor or using specific medication. The renal function of controls is presumed to be sufficient, as they have been approved for kidney donation.

‡Previous vascular surgery includes percutaneous coronary intervention, percutaneous transluminal angioplasty, coronary artery bypass grafting and endovascular aneurysm repair.

Table S4. Significantly differentially expressed proteins after in aortic SMC derived from non-diabetic AAA patients (ND-AAA)(n=19), diabetic AAA patients (D-AAA)(n=5) and aortic controls (C)(n=8).

No.	Gene	Log2(FC)	*P* value
1	PGP	∞	0,007
2	NUDT19	∞	0,009
3	RAD21	∞	0,011
4	MGP	∞	0,038
5	DNASE1L1	∞	0,040
6	FADD	∞	0,040
7	UPP1	∞	0,007
8	MAEA	∞	0,048
9	CHERP	∞	0,010
10	OXR1	∞	0,034
11	AFM	∞	0,017
12	SGCE	∞	0,047
13	FADS2	∞	0,018
14	RPLP1	∞	0,005
15	FABP3	∞	0,015
16	CFH	∞	0,024
17	ADRA2A	∞	0,047
18	DBT	∞	0,041
19	COL6A2	∞	0,024
20	IFITM1	∞	0,009
21	DSP	∞	0,030
22	NPR3	∞	0,012
23	GAP43	∞	0,013
24	DTYMK	∞	0,041
25	NUDT1	∞	0,021
26	SPTBN1	∞	0,024
27	IDI1	∞	0,012
28	ATP6AP1	∞	0,026
29	CYP1B1	∞	0,004

30	CERCAM	∞	0,008
31	DTX3L	∞	0,044
32	MMAB	∞	0,024
33	APIP	∞	0,024
34	FNBP1	∞	0,024
35	NAA50	∞	0,037
36	TBC1D17	∞	0,024
37	CMTM6	∞	0,003
38	UGGT2	∞	0,047
39	PDP1	∞	0,024
40	NTM	∞	0,022
41	CTSF	∞	0,042
42	FBLN5	∞	0,040
43	LEMD3	∞	0,026
44	ETFDH	23,545	0,002
45	SGSH	22,659	0,026
46	EIPR1	19,200	0,004
47	MYO18A	16,159	0,023
48	GBAS	16,023	0,014
49	CTSH	15,343	0,041
50	PBXIP1	11,548	0,043
51	FGL2	8,021	0,006
52	CPD	7,393	0,002
53	NME1	6,663	0,040
54	PFN2	5,809	0,021
55	RBMS1	5,582	0,023
56	SELENBP1	5,548	0,012
57	THSD4	4,682	0,016
58	PACSIN3	4,680	0,044
59	SCRN2	3,997	0,031
60	NQO2	3,297	0,046
61	NQO1	2,944	0,033

62	TMEM165	2,783	0,017
63	NDUFA4L2	2,532	0,021
64	PITRM1	2,513	0,009
65	LXN	2,512	0,029
66	AKR1C1	2,509	0,024
67	ABCC1	2,407	0,022
68	COL8A1	2,327	0,039
69	TIMP3	2,074	0,014
70	MAGT1	2,072	0,045
71	PGM2	2,000	0,020
72	ADAR	1,821	0,029
73	CDH11	1,815	0,040
74	TGFBI	1,804	0,029
75	CAT	1,803	0,036
76	EPHX1	1,677	0,018
77	PPL	1,606	0,050
78	EZR	1,590	0,043
79	PDLIM1	1,557	0,042
80	GYS1	1,421	0,034
81	ITGAV	1,410	0,039
82	HADHB	1,401	0,043
83	TAGLN2	1,392	0,003
84	MYL12A	1,322	0,023
85	TKT	1,320	0,008
86	DPYSL3	1,298	0,039
87	ATP1A1	1,256	0,024
88	ACTN1	1,198	0,032
89	FLNA	1,172	0,048
90	ACTG1	1,092	0,046

An log2(fold change (FC)) of ∞ signifies that the protein was found in one or two of the three study groups, but was absent in the others.

Table S5. Significantly differentially expressed proteins after applying a 50% data presence filter in aortic SMC derived from non-diabetic AAA patients (ND-AAA)(n=19) compared to aortic controls (C)(n=8).

No.	Gene Name	Expression in ND-AAA vs C	Log2(FC)	*P* value
1	PGP	L	-∞	0,002
2	UPP1	L	-∞	0,002
3	FGL2	L	-5,839	0,013
4	CPZ	L	-5,673	0,041
5	NUDT1	L	-4,813	0,026
6	MRPS36	L	-4,748	0,022
7	MRPL1	L	-3,965	0,050
8	CSTF3	L	-3,332	0,042
9	TRAPPC4	L	-3,181	0,034
10	SFRP1	L	-3,047	0,031
11	FABP3	L	-2,992	0,044
12	SLC30A1	L	-2,897	0,032
13	PAIP1	L	-2,568	0,022
14	LTBP1	L	-2,541	0,030
15	NDUFA4L2	L	-2,532	0,007
16	PITRM1	L	-2,513	0,007
17	ALDH2	L	-2,163	0,043
18	ADPRHL2	L	-2,113	0,036
19	MAGT1	L	-2,072	0,021
20	MTHFD1L	L	-1,968	0,045
21	ADAR	L	-1,821	0,013
22	PGM2	L	-1,816	0,009
23	PPL	L	-1,606	0,014
24	MAN2B2	L	-1,530	0,038
25	ADAMTS1	L	-1,518	0,026
26	GYS1	L	-1,421	0,010
27	GJA1	L	-1,408	0,022
28	EPHX1	L	-1,398	0,047

29	PCK2	L	-1,332	0,045
30	ACTN1	H	1,129	0,028
31	CNN2	H	1,247	0,029
32	MYL12A	H	1,284	0,020
33	TIMP3	H	1,622	0,041
34	CDH11	H	1,645	0,020
35	CERCAM	H	2,102	0,049
36	TP53I11	H	2,162	0,040
37	TMEM165	H	2,228	0,026
38	CPD	H	3,893	0,008
39	ALDH1B1	H	4,310	0,033
40	RBMS1	H	4,528	0,011
41	CTHRC1	H	6,063	0,028
42	CMTM6	H	∞	0,005

A negative log2(fold change (FC)) indicates the protein expression was lower (L) in ND-AAA-SMC compared to C-SMC, while a positive log2(FC) indicates that the protein expression was higher (H) in ND-AAA-SMC compared to C-SMC. Additionally, an log2(FC) of $-\infty$ or ∞ signifies that the protein was exclusively detected in the C-SMC or ND-AAA-SMC, respectively.

Table S6. Identifiers of the KEGG, Reactome and GO terms.

Term	Identifier
Actin binding	GO:0003779
Actin cytoskeleton organization	GO:0030036
Actin filament binding	GO:0051015
Actin filament bundle	GO:0032432
Actin filament bundle organization	GO:0061572
Arachidonic acid metabolism	R-HSA-2142753
Carbohydrate metabolic proc.	GO:0005975
Carbon metabolism	Path:hsa01200
Carboxylic acid metabolic proc.	GO:0019752
Cell adhesion	GO:0007155
Cell adhesion molecule binding	GO:0050839
Cell junction organization	R-HSA-446728
Cell-Cell communication	R-HSA-1500931
Cell-extracellular matrix interactions	R-HSA-446353
Cellular carbohydrate biosynthetic proc.	GO:0034637
Cellular response to chemical stress	R-HSA-9711123
Chemical carcinogenesis-DNA adducts	Path:hsa05204
Chemical carcinogenesis-reactive oxygen species	Path:hsa05208
Contractile fiber	GO:0043292
Cytoskeletal protein binding	GO:0008092
Dicarboxylic acid metabolic proc.	GO:0043648
Extracellular exosome	GO:0070062
Extracellular matrix organization	R-HSA-1474244
Extracellular region	GO:0005576
Fatty acid metabolic proc.	GO:0006631
Fatty acid metabolism	R-HSA-8978868
Focal adhesion	Path:hsa04510
Focal adhesion	GO:0005925
Glutathione metabolism	Path:hsa00480
Glycerol metabolic proc.	GO:0006071

Homotypic cell-cell adhesion	GO:0034109
Icosanoid metabolic proc.	GO:0006690
Identical protein binding	GO:0042802
Integrin binding	GO:0005178
KEAP1-NFE2L2 pathway	R-HSA-9755511
Lipid metabolic proc.	GO:0006629
MAPK signaling pathway	Path:hsa04010
MET activates PTK2 signaling	R-HSA-8874081
Metabolic pathways	Path:hsa01100
Metabolism	R-HSA-1430728
Metabolism of lipids	R-HSA-556833
Mitochondrial matrix	GO:0005759
Monocarboxylic acid metabolic proc.	GO:0032787
Non-integrin membrane-ECM interactions	R-HSA-3000171
Nuclear events mediated by NFE2L2	R-HSA-9759194
Oxidoreductase activity	GO:0016491
Oxidoreductase activity acting on the CH-OH group of donors NAD or NADP as acceptor	GO:0016616
Pentose phosphate pathway	Path:hsa00030
Pentose phosphate pathway	R-HSA-71336
Prostaglandin metabolic proc.	GO:0006693
Proteoglycan binding	GO:0043394
Reg. of actin cytoskeleton	Path:hsa04810
Sarcomere	GO:0030017
Small molecule metabolic proc.	GO:0044281
Structural constituent of synapse	GO:0098918
Supramolecular fiber	GO:0099512
Syndecan interactions	R-HSA-3000170
Tight junction	Path:hsa04530
Transmembrane transporter binding	GO:0044325
Unsaturated fatty acid metabolic proc.	GO:0033559
Xenobiotic metabolic proc.	GO:0006805

Contractile actin filament bundle	GO:0097517
Vesicle	GO:0031982

Table S7. Significantly differentially expressed proteins after applying a 75% data presence filter in aortic SMC derived from diabetic AAA patients (D-AAA)(n=5) compared to non-diabetic AAA patients (ND-AAA)(n=19).

No.	Gene	Expression in D-AAA vs ND-AAA	Log2(FC)	*P* value
1	CPD	L	-7,393	0,007
2	USP10	L	-3,451	0,050
3	UBXN6	L	-3,435	0,047
4	FAM160B1	L	-3,369	0,019
5	TMEM165	L	-2,783	0,029
6	PRKG1	L	-2,512	0,035
7	TIMP3	L	-2,074	0,019
8	ADAM10	L	-1,930	0,020
9	COL5A1	L	-1,769	0,026
10	COLGALT1	L	-1,606	0,032
11	EZR	L	-1,590	0,022
12	EML4	L	-1,563	0,030
13	PDLIM1	L	-1,557	0,018
14	LAMC1	L	-1,462	0,023
15	FN1	L	-1,459	0,036
16	TAGLN2	L	-1,392	0,002
17	MYL12A	L	-1,322	0,038
18	FLNB	L	-1,290	0,011
19	FLNC	L	-1,271	0,026
20	HIST1H2BM	L	-1,237	0,047
21	ACTN1	L	-1,198	0,033
22	FLNA	L	-1,172	0,025
23	PDIA3	L	-1,137	0,049
24	ACTG1	L	-1,092	0,018
25	G6PD	H	1,242	0,026
26	ATP1A1	H	1,256	0,017
27	CBR1	H	1,273	0,046

28	DPYSL3	H	1,298	0,008
29	TKT	H	1,320	0,003
30	AKR1B1	H	1,378	0,020
31	LAP3	H	1,387	0,049
32	HADHB	H	1,401	0,031
33	XPNPEP1	H	1,452	0,047
34	PGD	H	1,523	0,047
35	ACAT1	H	1,547	0,037
36	ADK	H	1,611	0,046
37	SQOR	H	1,648	0,039
38	EPHX1	H	1,677	0,019
39	ARSA	H	1,706	0,024
40	TGFBI	H	1,804	0,015
41	PTGS1	H	1,839	0,026
42	GSTM1	H	2,122	0,019
43	DUT	H	2,182	0,045
44	PITRM1	H	2,214	0,037
45	VPS26B	H	2,307	0,032
46	ABCC1	H	2,407	0,006
47	AKR1C1	H	2,509	0,011
48	LXN	H	2,512	0,009
49	IGFBP6	H	2,543	0,039
50	SCRN2	H	2,737	0,025
51	SFRP4	H	2,838	0,032
52	ITSN1	H	2,905	0,023
53	NQO1	H	2,944	0,019
54	LAMA4	H	3,330	0,006
55	MTX2	H	3,874	0,027
56	PACSIN3	H	4,680	0,016
57	SDSL	H	4,868	0,023
58	ALG2	H	5,022	0,035
59	MYO5A	H	5,193	0,035

60	PFN2	H	5,809	0,007
61	FGL2	H	8,021	0,008
62	EIPR1	H	19,200	0,001
63	ETFDH	H	23,545	0,001

A negative log2(fold change (FC)) indicates the protein expression was lower (L) in D-AAA-SMC compared to ND-AAA-SMC, while a positive log2(FC) indicates that the protein expression was higher (H) in D-AAA-SMC compared to ND-AAA-SMC. Additionally, an log2(FC) of $-\infty$ or ∞ signifies that the protein was exclusively detected in the ND-AAA-SMC or D-AAA-SMC, respectively.
